# Dolphyin: a combinatorial algorithm for identifying 1-Dollo phylogenies in cancer

**DOI:** 10.1186/s13015-026-00303-2

**Published:** 2026-06-08

**Authors:** Daniel W. Feng, Mohammed El-Kebir

**Affiliations:** 1 Department of Biomedical Informatics, Harvard Medical School, Boston, MA USA; 2 Siebel School of Computing and Data Science, University of Illinois Urbana-Champaign, Urbana, IL USA; 3 Cancer Center at Illinois, University of Illinois Urbana-Champaign, Urbana, IL USA

**Keywords:** Intra-tumor heterogeneity, Persistent perfect phylogeny, Consecutive ones property, Combinatorics

## Abstract

**Background:**

Several recent cancer phylogeny inference methods have used the *k*-Dollo evolutionary model for single-nucleotide variants, which requires a phylogeny *T* on binary sequencing data matrix *B* such that each variant is gained once and lost at most *k* times. The 1-Dollo variant has been studied extensively but its hardness remains open.

**Results:**

We prove that the 1-Dollo Linear Phylogeny (1DLP) problem, where we additionally require the resulting 1-Dollo phylogeny *T* to be linear, is equivalent to verifying whether matrix *B* has the Consecutive Ones Property, which can be determined in polynomial time. We also show that some practical extensions of 1DLP, such as the minimization of false negatives, are NP-hard. We then show how to recursively decompose any 1-Dollo phylogeny *T*, not necessarily linear, into several 1-Dollo linear phylogenies and extend this characterization to all matrices *B* that admit 1-Dollo phylogenies. We use this characterization to develop Dolphyin, a new exponential-time algorithm for inferring 1-Dollo phylogenies. Dolphyin is runtime-competitive with integer linear programming-based algorithm SPhyR (El-Kebir 2018) on simulated datasets and infers 1-Dollo phylogenies with false negative sequencing error rates at or below simulated ground truth rates. We apply Dolphyin to acute myeloid leukemia datasets and find that the majority of the cancers can be explained by 1-Dollo phylogenies with error rates in line with the used sequencing technology.

**Conclusion:**

Our work develops a novel, combinatorial algorithm for practical inference of 1-Dollo phylogenies.

## Background

The clonal theory of cancer states that tumors are composed of heterogeneous clones, which are groups of cells with similar genotypes. Clones arise from an evolutionary process during which somatic mutations accumulate in cell populations [1]. By performing bulk or single-cell sequencing on these clones’ DNA or RNA, scientists can identify common somatic mutations such as single-nucleotide variants (SNVs), copy number aberrations (CNAs), or structural variants (SVs). Then, algorithms attempt to infer phylogenies, which represent the evolution of the tumor, from sequencing data for downstream analysis and clinical decision-making [2]. These phylogenetic inference algorithms utilize sequencing-technology specific error characteristics and constraints on how these somatic mutations accumulate, which constitute an evolutionary model specific to the somatic mutations of interest [3].

In this work, we focus on the presence or absence of SNVs in single-cell DNA sequencing data. More precisely, we are given single-cell data in the form of a matrix *B* where each row in the data is a taxon, representing a tumor cell, and each column is a single-nucleotide variant (SNV), hereafter referred to as a character. The entries in the data matrix would then be either 0 or 1, indicating the absence or presence of a mutation in a particular cell. We wish to find a phylogeny, i.e. a rooted, node-labeled tree *T* whose leaves represent the extant cells of the tumor, internal nodes represent ancestral tumor cells, and the root represents a normal cell [1], that explains this data. We would then need to assume an evolutionary model that constrains the phylogenies allowed on this data. Under the well-studied two-state perfect phylogeny model [4–6], for example, no character can be lost once gained in a path starting from the root of tree *T*. Detecting whether an assumed error-free binary data matrix allows a phylogeny under the perfect phylogeny model is solvable in polynomial time [4, 7].

However, the restrictiveness of the perfect phylogeny model of evolution has inspired investigation into a wide range of more generalized and biologically-plausible models [8, 9]. Many analyses have operated under the flexible *k*-Dollo model of evolution, under which any character may be gained exactly once but lost in the tumor’s evolution at most *k* times [10–13]. This flexibility affords the incorporation of common biological events, such as CNAs that may delete previously gained SNVs, and can thus be much more realistically versatile in biological analysis. The ∞-Dollo phylogeny inference [14, 15] and tree size-constrained versions of Dollo phylogeny inference [14] are known to be NP-hard. Contrastingly, the problem variant for Dollo inference where the total number of losses summed over all characters is minimized, rather than outright bounded per character, can be solved in polynomial time when the resulting phylogeny is clade-constrained, that is, every clade (leafset of a subtree) in the inferred phylogeny must belong to a user-supplied set of allowed clades [10].

**Fig. 1:**
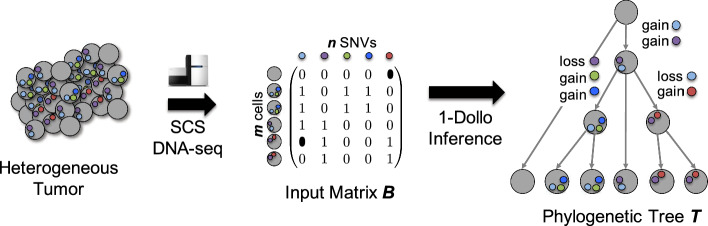
Tumor phylogeny estimation from single-cell sequencing (SCS) data under the 1-Dollo, or persistent phylogeny, model of evolution. Heterogeneous tumors are composed of distinct cellular populations with distinct complements of somatic mutations, including single-nucleotide variants (SNVs). During cancer progression, SNVs are frequently lost due to copy-number aberrations, but rarely introduced more than once. Here, single-cell sequencing of a tumor yields an input matrix *B*, whose *m* rows are individual cells and *n* columns are SNVs. Under the 1-Dollo evolutionary model, each SNV can only be gained once and lost once. Our goal is to infer a satisfying phylogeny *T* under this model or demonstrate that one does not exist

A subcase of the *k*-Dollo problem is the k=1 subcase or 1-Dollo problem, also known as the persistent phylogeny problem (Fig. 1). The 1-Dollo problem has been extensively studied, using various problem statements, for over 20 years [16]. For example, characterizations of the 1-Dollo problem have yielded an exact algorithm that solves the 1-Dollo problem in time polynomial to the number of taxa and exponential to the number of characters [17]. Other work has also developed Integer Linear Programming (ILP) solutions to the 1-Dollo problem and shown a connection between galled trees and 1-Dollo phylogenies [18]. Graph-based approaches, specifically the ability to manipulate colored graphs representative of data matrices using sequenced and specific graph operations, have additionally yielded polynomial-time algorithms for a restricted version of the 1-Dollo problem [19]. However, the complexity of the general 1-Dollo problem remains an open question [19].

Separately, tumor phylogenies can either be linear—that is, phylogeny *T* has no nontrivial branching points in which cell evolution diverges—or branching otherwise, based on the tumor’s selective pressures. In this work, we define “branching points” to entail internal nodes in *T* with more than one child that are also internal nodes. Many phylogenies on real data, while branching, have exhibited a disproportionately small number of branching points relative to the number of taxa [11, 20]. Such phylogenies plausibly arise when the tumor microenvironment exerts severe enough selective pressure to limit branching to a few, highly viable offshoot clones [21]. This observation motivates phylogenetic inference that is specialized for finding linear or near-linear phylogenies. For example, machine learning techniques have been used to determine if a tumor phylogeny is likely linear [22] and, in previous work, we showed that determining the minimum number of changes from 0 to 1 in a data matrix such that the altered matrix is then representative of even a linear perfect phylogeny is NP-hard [23]. However, as far we are aware, determining 1-Dollo phylogenies on data that are strictly linear has not yet been explicitly examined.

In this paper, our aims are first theoretical and second experimental. First, we draw an equivalence between determining if a data matrix *B* admits a 1-Dollo linear phylogeny and determining if *B* has the consecutive ones property, which is a known property verifiable in polynomial time [24]. We use this theoretical characterization of matrices admitting 1-Dollo linear phylogenies to discuss natural problem variants, such as determining 1-Dollo linear phylogenies with fixed character-state vectors for the root or terminating leaf, and show that determining the minimal number of false negative entries in a sequencing data input to allow such a phylogeny, contrastingly, is NP-hard. As a tree can be recursively decomposed around its branching points, we then use this linear subcase of the 1-Dollo problem to recursively characterize all matrices admitting any 1-Dollo phylogeny, regardless of branching, with a series of necessary and sufficient conditions. Second, we develop a combinatorial algorithm, Dolphyin (DOllo Linear PHYlogeny INference Method), that uses this theoretical characterization to practically determine 1-Dollo phylogenies on sequencing data. We show that Dolphyin, which relies on determining linear chains of taxa satisfying the 1-Dollo model of evolution and then recursing on remaining taxa, is runtime-competitive with SPhyR—an ILP-based method of inference for the *k*-Dollo problem. Additionally, we adapt Dolphyin to probabilistically correct for false-negative errors in sequencing. We apply Dolphyin to simulated datasets with false-negative errors and show that Dolphyin yields 1-Dollo phylogenies with inferred rates of error at or below the true rate of error. Finally, we apply Dolphyin to 99 real datasets of acute myeloid leukemia (AML) single cell sequencing data [20] and find that Dolphyin infers 1-Dollo phylogenies for 55 datasets with false negative error rates consistent with the used sequencing technology.

## Methods

### Problem statement

Suppose we have sequenced *m* cells of a tumor and identified *n* single-nucleotide variants (SNVs). We are given a binary matrix $B \in \{0,1\}^{m \times n}$, whose *m* rows or *taxa* correspond to the sequenced cells and whose *n* columns or *characters* correspond to the SNVs. The tumor cells share a common evolutionary history, represented by a rooted and node-labeled tree *T* such that the leaves of *T* specifically correspond to these cells. Here, we require *T* to adhere to the *k*-Dollo evolutionary model [9], defined as follows.

#### Definition 1

A rooted, node-labeled tree *T* is a *k*-*Dollo phylogeny for an* m×n *binary matrix* $B = [b_1,\ldots ,b_m]^\top$ *rooted at* $b_0=[b_{0,1},\ldots ,b_{0,n}]^\top$ provided

1. each node *v* in *T* is labeled by a binary vector $b_T(v) = [b(v,1),\ldots ,b(v,n)]^\top$;
2. the root *r* of *T* is labeled by $b_T(r)=b_0$;
3. *T* has *m* leaves such that each taxon t∈[m] corresponds to exactly one leaf $\sigma _T(t) = v$ in *T* with parent *u* such that $b_T(v) = b_T(u) = b_t$;
4. for each character c∈[n] where $$b_{0,c} = 0$$, there is at most one *gain edge* (*u*, *v*) such that $$b_T(u,c) = 0$$ and $$b_T(v, c) = 1$$, and at most *k* *loss edges* $\left(u^{\prime },v^{\prime }\right)$ such that $$b_T(u', c) = 1$$ and $$b_T(v', c) = 0$$;
5. for each character c∈[n] where $b_{0,c} = 1$, there is no gain edge and at most *k* loss edges.

We omit the subscript *T* from node labeling $b_T(v)$ and taxa mapping $\sigma _T(t)$ if it is clear from context that they apply to a particular tree *T*. In this paper, we restrict our attention to the common case where at most k=1 loss per character is allowed and, unless otherwise stated, assume that the root must be labeled by all 0s, i.e. $b(r)=b_0=0$. We call such trees simply 1-Dollo phylogenies for *B*. Thus, we seek to solve the following problem.

#### Problem 1

(1-Dollo Phylogeny (1DP) *Problem*) Given binary matrix $B \in \{0,1\}^{m \times n}$, build a 1-Dollo phylogeny *T* for *B* or show that one does not exist.

We note that the above problem is also known as the persistent phylogeny problem [17]. In addition to the above problem, we are also interested in the problem where *T* is required to be a 1-*Dollo linear phylogeny for* *B*.

#### Definition 2

A 1-Dollo phylogeny for a binary matrix $B\in \{0,1\}^{m \times n}$ is *linear* if the removal of the *m* leaves of *T* corresponding to the *m* taxa yields a chain graph.

Note that this characterization hinges on the condition in Definition 1 that all leaves’ labels must match their parents’ labels, since this guarantees that all gain and loss edges must necessarily be internal.

#### Problem 2

(1-Dollo Linear Phylogeny (1DLP) *Problem*) Given binary matrix $B \in \{0,1\}^{m \times n}$, is there a 1-Dollo linear phylogeny *T* for *B*, and if so, build one.

### Combinatorial characterization

We characterize the solution spaces of both 1DP and 1DLP, starting with the more restrictive problem, 1DLP. We then build on this result by discussing the complexity of common problem variants to 1DLP. Then, we demonstrate that solutions to the 1DP problem can be recursively characterized in terms of itself and 1-Dollo linear phylogenies. We delegate all proofs to the appendix.

To simplify the exposition, we introduce the following definition of a *compact* phylogeny and lemma, stating that one can assume without loss of generality that all internal, non-root nodes of solutions *T* must either correspond to observed taxa or are branching points, and all internal edges must be either a gain or a loss edge for some character. Intuitively, any internal, non-root nodes of a given 1-Dollo phylogeny $T^{\prime }$ that do not correspond to one of these two cases can always be contracted to create a compact 1-Dollo phylogeny *T*.

#### Definition 3

A 1-Dollo phylogeny *T* with root *r* for a matrix $B \in \{0,1\}^{m \times n}$ is *compact* if

1. each internal node u≠r of *T* either corresponds to an *observed taxon*, i.e. *u* has a leaf child *v* such that $b_T(u) = b_T(v)$; or is a *branching point*, i.e. *u* has ℓ>1 distinct outgoing edges $(u,v_1),\ldots ,(u,v_{\ell })$ such that $b_T(u) \ne b_T(v_1), \ldots , b_T(u) \ne b_T(v_{\ell })$; and
2. and every internal edge (*u*, *v*) is either a gain or loss edge for some character so that $b_T(u) \ne b_T(v)$.

#### Lemma 1

For any 1-Dollo phylogeny *T* for a matrix *B*, there exists a unique compact 1-Dollo phylogeny $T^{\prime }$, obtainable from *T*, for *B*.

#### 1DLP and the Consecutive Ones Property

We show that the 1DLP problem is equivalent to determining whether the input binary matrix *B* satisfies the consecutive ones property, which is defined as follows.

##### Definition 4

(*Ref.* [24]) An m×n binary matrix *B* has the *consecutive ones property* (C1P) if there exists a permutation $\pi : [m] \rightarrow [m]$ such that for each column *c* the 1s appear consecutively when permuting the rows of *B* according to π.

**Fig. 2:**
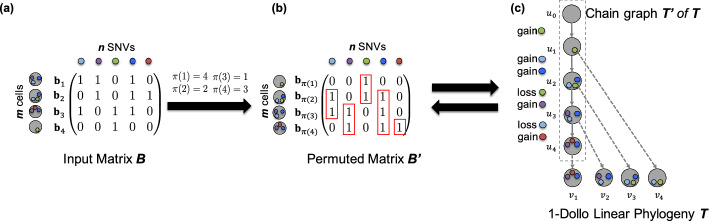
**a** An example matrix *B* demonstrating that the 1DLP problem is equivalent to the C1P problem. **b** Determining that *B* is C1P with permutation $$\pi : [m] \rightarrow [m]$$ yields permuted matrix $B^{\prime }$ such that the 1s are consecutive in each column. (c) This allows the construction of a 1-Dollo linear phylogeny *T* for *B* following Definition 5

To demonstrate this equivalence, we first propose the following construction of obtaining a tree *T* from a matrix *B* that is C1P with permutation π, illustrated in Fig. 2.

##### Definition 5

The rooted, node-labeled tree T(B,π) resulting from a binary matrix $B = [b_1,\ldots ,b_m]^\top$ that is C1P with permutation $\pi : [m] \rightarrow [m]$ has (i) a root node $r = u_0$ labeled by b(r)=0, (ii) internal nodes $u_1,\ldots ,u_m$ and leaves $v_1,\ldots ,v_m$ labeled by $b(u_t) = b(v_t) = b_{\pi (t)}$ for each taxon t∈[m], (iii) edges $(u_{t-1},u_t)$ and $(u_t,v_t)$ for each taxon t∈[m] and taxon leaf labeling $\sigma (v_t) = t$ for each taxon t∈[m].

This construction leads us to the main theorem of this section.

##### Theorem 1

There exists a 1-Dollo linear phylogeny *T* for *B* if and only if *B* is C1P.

As determining whether any binary matrix $B \in \{0,1\}^{m \times n}$ is C1P including determining the corresponding permutation $\pi : [m] \rightarrow [m]$ of rows is solvable in *O*(*mn*) time using PQ trees [25], 1DLP is similarly solvable in *O*(*mn*) time.

##### Corollary 1

1DLP is solvable in *O*(*mn*) time.

#### Rooted and terminating variants of 1DLP

A natural generalization of 1DLP is the Rooted 1-Dollo Linear Phylogeny (R1DLP) problem, where the root node *r* must be labeled by a given vector $b_0 \in \{0,1\}^{n}$ not necessarily equal to 0.

##### Problem 3

(Rooted 1-Dollo Linear Phylogeny (R1DLP) *Problem*) Given binary matrix $B \in \{0,1\}^{m \times n}$ and binary vector $b_0 \in \{0,1\}^n$, is there a 1-Dollo linear phylogeny *T* for *B* rooted at $b_0$, and if so, build one.

**Fig. 3:**
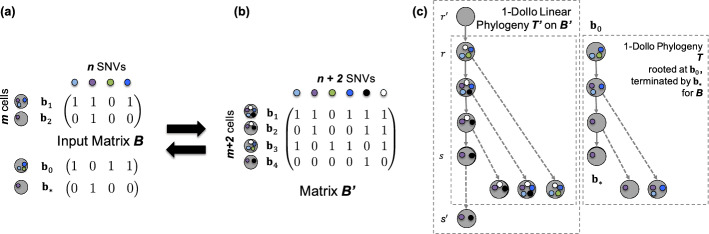
RT1DLP can be solved with a transformation of 1DLP. **a** An example matrix *B* with root vector $$b_0$$ and terminating state $$b_*$$. **b** Modified matrix $B^{\prime }$ derived from the transformation described in Definition 8 on *B*, $$b_0$$, and $$b_*$$. **c** 1-Dollo linear phylogeny $T^{\prime }$ for $B^{\prime }$, with corresponding 1-Dollo linear phylogeny *T* rooted at $$b_0$$ and terminating at $$b_*$$ for matrix *B*

Clearly, the 1DLP problem is a special case of the R1DLP problem where $b_0 = 0$. In the following, we show that the R1DLP problem can also be solved in *O*(*mn*) time by extending matrix $B = [b_{t,c}]$ as follows (Fig. 3).

##### Definition 6

The (m+1)×(n+1) binary matrix $$B'(B,b_0)$$ resulting from m×n binary matrix $$B = [b_{t,c}]$$ and *n*-dimensional binary vector $$b_0 = [b_{0,c}] $$ has entries $$b'_{t,c}$$ equal to

1 $$\begin{aligned} b'_{t,c} = {\left\{ \begin{array}{ll} b_{t,c}, &  \text{ if } t \in [m] \text{ and } c \in [n],\\ 1, &  \text{ if } t \in [m] \text{ and } c=n+1,\\ b_{0,c}, &  \text{ if } t = m+1 \text{ and } c \in [n],\\ 0, &  \text{ if } t=m+1 \text{ and } c=n+1.\\ \end{array}\right. } \end{aligned}$$

##### Lemma 2

There exists a 1-Dollo linear phylogeny *T* for *B* rooted at $$b_0$$ if and only if there exists a 1-Dollo linear phylogeny $T^{\prime }$ for $$B'(B,b_0)$$.

##### Corollary 2

R1DLP is solvable in *O*(*mn*) time.

A second generalization of 1DLP is the Rooted, Terminated 1-Dollo Linear Phylogeny (RT1DLP) problem where upon removal of the *m* leaves corresponding to the *m* taxa of *B* the root node *r* is labeled by $b_0$ and the sink node *s* is labeled by $b_*$ (Fig. 3). More precisely, we have the following definition for such a constrained 1-Dollo linear phylogeny.

##### Definition 7

A 1-Dollo linear phylogeny *T* for $B \in \{0,1\}^{m \times n}$ rooted at $b_{0}$ *terminates* at $b_{*}$ if removing the leaves $v_1,\ldots ,v_m$ corresponding to the *m* taxa yields a chain graph terminating at node *s* such that $b(s)=b_*$.

##### Problem 4

(Rooted, Terminated 1-Dollo Linear Phylogeny (RT1DLP) Problem) Given binary matrix $B \in \{0,1\}^{m \times n}$ and binary vectors $b_0,b_* \in \{0,1\}^n$, is there a 1-Dollo linear phylogeny *T* for *B* rooted at $b_0$ and terminating at $b_*$, and if so, build one.

Again, R1DLP (Problem 3) is a special case of RT1DLP where $b_*=0$ and 1DLP (Problem 2) is a special case of RT1DLP where $b_0=b_*=0$. The RT1DLP problem can be solved in *O*(*mn*) time by a similar matrix extension to *B* as discussed regarding R1DLP.

##### Definition 8

The (m+2)×(n+2) binary matrix $B'(B,b_0, b_*)$ resulting from m×n binary matrix $B = [b_{t,c}]$ and *n*-dimensional binary vectors $b_0 = [b_{0,c}]$ and $b_* = [b_{*,c}]$ has entries $b'_{t,c}$ equal to

2 $$\begin{aligned} b'_{t,c} = {\left\{ \begin{array}{ll} b_{t,c}, &  \text{ if } t \in [m] \text{ and } c \in [n],\\ 1, &  \text{ if } t \in [m] \text{ and } c = n+1,\\ 1, &  \text{ if } t \in [m] \text{ and } c = n+2,\\ b_{0,c}, &  \text{ if } t = m+1 \text{ and } c \in [n],\\ 0, &  \text{ if } t = m+1 \text{ and } c = n+1,\\ 1, &  \text{ if } t = m+1 \text{ and } c = n+2,\\ b_{*,c}, &  \text{ if } t = m+2 \text{ and } c \in [n],\\ 1, &  \text{ if } t = m+2 \text{ and } c = n+1,\\ 0, &  \text{ if } t = m+2 \text{ and } c = n+2. \end{array}\right. } \end{aligned}$$

##### Lemma 3

There exists a 1-Dollo linear phylogeny *T* for *B* rooted at $$b_0$$ and terminating at $$b_*$$ if and only if there exists a 1-Dollo linear phylogeny $T^{\prime }$ for $$B'(B,b_0,b_*)$$.

##### Corollary 3

RT1DLP is solvable in *O*(*mn*) time.

#### Additional variants of 1DLP

The direct equivalence from 1DLP to the Consecutive Ones Property allows several known properties from the latter to apply to 1DLP. For example, allowing for false negatives, a typical phenomenon in single-cell DNA sequencing due to allelic dropout [26], yields the following problem.

##### Problem 5

(Minimum Error 1-Dollo Linear Phylogeny (ME1DLP) Problem) Given binary matrix $$B \in \{0,1\}^{m \times n}$$ for *m* cells and *n* SNVs, determine the minimum number of 0 to 1 replacements in *B* such that the resulting matrix $B^{\prime }$ has a 1-Dollo linear phylogeny *T*.

As the equivalent problem of determining the minimum number of 0-to-1 matrix modifications of any binary matrix *B* to satisfy C1P is NP-hard [27], ME1DLP is also NP-hard.

##### Corollary 4

ME1DLP is NP-hard.

In a similar vein, the 1DLP problem variant with at most two gains and at most two losses per character is equivalent to the C1P generalization (2,∞) observed in [28], which is NP-hard.

##### Corollary 5

Determining whether a binary matrix $B \in \{0,1\}^{m \times n}$ admits a 1-Dollo linear phylogeny with at most two gains and at most two losses per character is NP-hard.

#### Recursive characterization of 1DP

We begin by stating the rooted version of 1DP (Problem 1), which has no linear constraint.

##### Problem 6

(Rooted 1-Dollo Phylogeny (R1DP) Problem) Given binary matrix $B \in \{0,1\}^{m \times n}$ and binary vector $b_0 \in \{0,1\}^n$, construct a 1-Dollo phylogeny *T* for *B* rooted at $b_0$ or determine that one does not exist.

In this section we will show how a 1DP problem instance $B \in \{0,1\}^{m\times n}$ can be recursively decomposed into smaller Rooted 1-Dollo Linear Phylogeny (R1DLP, Problem 3), Rooted, Terminated 1-Dollo Linear Phylogeny (RT1DLP, Problem 4) and Rooted 1-Dollo Phylogeny (R1DP, Problem 6) instances on submatrices of *B*.

To that end, we introduce the notation *B*[*X*, *C*] to indicate the submatrix of *B* induced by rows/taxa X⊆[m] and columns/characters C⊆[n]. Moreover, we define b⊘C to be the restriction of binary vector $b \in \{0,1\}^n$ to only characters C⊆[n]. For example, the restriction of $b = [0, 1, 1, 1, 1]^\top$ to characters $C=\{1,3\}$ equals $b \oslash C = [0,1]^\top$. We use the shorthand T⊘C to indicate that all node labels of *T* have been restricted to *C*, i.e. b(v)⊘C for all nodes *v* of *T*.

#### Recursive characterization of rooted 1-Dollo phylogenies

Like every tree structure, a 1-Dollo phylogeny *T* for a given binary matrix *B* can be recursively characterized. Key to the characterization are *branching points*, which are internal nodes with more than one non-leaf child. Let $v_*$ be the first branching point encountered by a tree traversal on *T* starting from its root *r* labeled by $b_0$. Let $v_*$ be labeled by $b_{*}$ and have $\ell \ge 2$ non-leaf children. If no such node exists then *T* is simply a 1-Dollo linear phylogeny for *B*, corresponding to the base case of the recurrence.

If there exists a branching point $v_*$ then, on the tree traversal from *r* to $v_*$, we encounter a subset $X_0 \subseteq [m]$ of taxa as well as identify sets $C^+_0, C^-_0 \subseteq [n]$ of characters that were gained or lost solely on this traversal, respectively. Note that a character first gained and then lost on this traversal is present in both $C^+_0$ and $C^-_0$. Also, note that $C^-_0$ may not be a subset of $C^+_0$; for instance, there may be a character *c* that was previously gained such that $b_{0,c}=1$ that is subsequently lost prior to the branching point $v_*$, leading to $c \not \in C^+_0$ and $c \in C^-_0$. Let $C_0 = C^-_0 \cup C^+_0$, and let $v_{*}$ be labeled by binary vector $b_{*}$. The encountered nodes on the traversal from *r* to $v_*$ induce a subtree $T_0$ such that its restriction $T_0 \oslash C_0$ is precisely a 1-Dollo linear phylogeny for submatrix $B[X_0, C_0]$ rooted at $b_{0} \oslash C_0$ and terminating at $b_* \oslash C_0$. To characterize the remainder of the tree, observe that performing a traversal of *T* starting at the *i*-th outgoing edge from $v_*$ yields a tree $T_i$ composed of taxa $X_i$, gained characters $C^+_i$ and lost characters $C^-_i$. Let $C_{i} = C^-_i \cup C^+_i$. We have that $T_i \oslash C_{i}$ is precisely a 1-Dollo phylogeny for submatrix $B[X_i,C_i]$ rooted at $b_{*} \oslash C_{i}$, stemming from the intuition that decomposing a tree cannot add new unique edges or nodes to the sum of its parts.

##### Lemma 4

For a given binary vector $b_0 \in \{0,1\}^n$ and 1-Dollo phylogeny *T* for matrix $B \in \{0,1\}^{m \times n}$, let $T_0$ be the subtree of *T* obtained by traversing from the node $v_0$ labeled by $b_0$ to a first branching point $v_*$ with label $b_*$, and let $T_1,\ldots ,T_\ell$ be the subtrees of *T* obtained by traversing along each of the $\ell \ge 2$ outgoing edges from $v_*$. Let $C^+_i$, $C^-_i$ and $X_i$ be the gained characters, lost characters and observed taxa, respectively, in tree $T_i$ where $i \in \{0,\ldots ,\ell \}$. Let $C_i = C^-_i \cup C^+_i$ for all $i \in \{0,\ldots ,\ell \}$. Then, the following conditions hold.

1. Sets $C^+_{0},\ldots ,C^+_{\ell }$ are pairwise disjoint, sets $C^-_{0},\ldots ,C^-_{\ell }$ are pairwise disjoint, and sets $C_{1},\ldots ,C_{\ell }$ are pairwise disjoint.
2. Sets $X_0,\ldots ,X_\ell$ are pairwise disjoint, and $X_{0} \cup \ldots \cup X_\ell$ is the set of all taxa observed in the subtree of *T* rooted at $v_0$.
3. $$C^+_i \subseteq [n] \setminus C^-_0$$ for all $$i \in [\ell ]$$.
4. Tree $T_0 \oslash C_0$ is a 1-Dollo linear phylogeny for submatrix $B_{0} = B[X_0,C_0]$ rooted at $b_{0} \oslash C_0$ terminating at $b_* \oslash C_0$.
5. For each $i \in [\ell ]$, tree $T_i \oslash C_i$ is a 1-Dollo phylogeny for submatrix $B_{i} = B[X_i,C_i]$ rooted at $b_{*} \oslash C_i$.

Thus, at each branching point $v_*$ of *T* with $\ell \ge 2$ non-leaf children, we obtain a single instance of Rooted, Terminated 1-Dollo Linear Phylogeny (RT1DLP, Problem 4) and ℓ instances of Rooted 1-Dollo Phylogeny (R1DP, Problem 6). Each of these ℓ instances can be further decomposed in a recursive fashion by identifying subsequent branching points.

#### Recursive decomposition of matrix *B*

The above recursive decomposition of a given 1-Dollo phylogeny *T* for matrix *B* rooted at some $b_0$ yields trees $T_{0}, T_{1}, \ldots , T_{\ell }$ on submatrices $B_{0}, B_{1}, \ldots , B_{\ell }$, respectively. As a phylogeny *T* can be decomposed, therefore, matrix *B* can also be decomposed. However, it is far less apparent how to do so without prior knowledge of the 1-Dollo phylogeny *T* on *B*. Here, we describe how given *B* and $b_0$, submatrices $B_{0} = B[X_0, C_0], \ldots , B_{\ell } = B[X_\ell , C_\ell ]$ can be inferred solely from the taxa set $X_{0} \subseteq [m]$ and the two character sets $C^-_0, C^+_{0} \subseteq [n]$. We begin by noting how $b_0$ and character sets $C^-_0, C^+_0$ uniquely determine the label of a potential branching point, since there is a unique path from the root to the branching point containing gains $C^+_0$ and losses $C^-_0$. To that end, we define the following function.

##### Definition 9

Given binary vector $b_0=[b_{0,1},\ldots ,b_{0,n}]^\top$ and characters $C^-, C^+ \subseteq [n]$, the *n*-dimensional binary vector $b(b_0,C^+,C^-) = [b_1,\ldots ,b_n]^\top$ consists of entries

3 $$\begin{aligned} b_c = {\left\{ \begin{array}{ll} 0, &  \text{ if } c \in C^-,\\ 1, &  \text{ if } c \in C^+ \setminus C^-,\\ b_{0,c}, &  \text{ otherwise. } \end{array}\right. } \end{aligned}$$

##### Lemma 5

Let *T* be a 1-Dollo phylogeny for matrix *B*. For any node $v_0$ labeled by $b_0$ and descendant node $v_*$ labeled by $b_*$ it holds that $b_* = b(b_0,C^+,C^-)$ where $C^+$ and $C^-$ are the characters that are gained and lost, respectively, on the path from $v_0$ to $v_*$.

Knowledge of sets $X_{0}$, $C^+_{0}$, and $C^-_{0}$ immediately implies knowledge of $B_{0} = B[X_0, C_0]$ since $C_0 = C^+_{0} \cup C^-_{0}$. Additional knowledge of $b_0$ allows us to infer the terminal label $b_* \oslash C_0 = b(b_{0},C^+_0,C^-_0)\oslash C_0$ of RT1DLP instance $(B_0,b_0 \oslash C_0, b_* \oslash C_0)$.

Thus, our only nontrivial goal is to infer submatrices $B_{1} = B[X_1, C_1], \ldots , B_{\ell } = B[X_\ell , C_\ell ]$ defined by taxa $X_1,\ldots ,X_\ell$ and characters $C_1,\ldots ,C_\ell$. We note that by the definition of a 1-Dollo phylogeny, only characters $c \in [n] \setminus C^-_{0}$ can be potentially gained or lost after the branching point labeled by $b_*$, and each such *c* can only be gained and potentially lost in specifically one tree $T_i$. To detail whether any character *c* must be gained or lost in 1-Dollo phylogeny $T_i$ on some proposed matrix $B_i$, we provide the following definition.

##### Definition 10

A character c∈[n] is *variable* w.r.t. an m×n matrix $B = [b_{t,c}]$ and *n*-dimensional vector $b = [b_{c}]$ if there exists a taxon t∈[m] such that $b_{t,c}\ne b_{c}$.

Our goal therefore translates into determining a partition $\{X_{1}, \ldots , X_{\ell }\}$ of $[m] \setminus X_{0}$ and partition $\{C_{1} ,\ldots , C_{\ell }\}$ of characters $C_{*} = [n] \setminus C^-_{0}$ such that each character $c \in C_{*}$ is variable w.r.t. at most one submatrix $B_i$ and $b_*$ (where $i \in [\ell ]$). To that end, we define the following matrix $\bar{B}(B,b_*, X_0, C_*)$.

##### Definition 11

The $(m - |X_0|) \times |C_*|$ *complement matrix* $\bar{B}(B,b_*, X_0, C_*)$ obtained from m×n binary matrix $B = [b_{t,c}]$ and *n*-dimensional vector $b_* = [b_{*,c}]$ has entries

4 $$\begin{aligned} \bar{b}_{t,c} = {\left\{ \begin{array}{ll} b_{t, c}, &  \text{ if } b_{*,c} = 0,\\ 1 - b_{t, c}, &  \text{ if } b_{*,c} = 1,\\ \end{array}\right. } \end{aligned}$$

where $$t \in [m] \setminus X_0$$ and $$c \in C_*$$.

##### Lemma 6

Let $\bar{B}_i$ be a submatrix of $\bar{B}(B,b_*,X_0,C_*)$ defined by characters $C_i \subseteq C_*$ and taxa $X_i \subseteq [m] \setminus X_0$ and let $B_i = B[X_i,C_i]$. Then, character $c \in C_i$ is variable w.r.t. $(B_i,b_*)$ if and only if $\bar{B}_i$ contains a 1 in column *c*.

Whether a character is variable with respect to vector $b_{*}$ and submatrix $B_i$ thus corresponds to whether the character column in $\bar{B}_i$ contains a 1. Since any character must be variable in at most one submatrix, our goal now finally equates to inferring a *block diagonal matrix decomposition* into block matrices $\bar{B}_1 = \bar{B}[X_1, C_1],\ldots ,\bar{B}_\ell = \bar{B}[X_{\ell }, C_{\ell }]$ of $\bar{B}(B,b_*,X_0,C_*)$, i.e.

5 $$\begin{aligned} \bar{B} = \begin{pmatrix} \bar{B}_1 &  0 &  \cdots &  0\\ 0 &  \bar{B}_2 &  \cdots &  0\\ \vdots &  \vdots &  \ddots &  \vdots \\ 0 &  0 &  \cdots &  \bar{B}_\ell \end{pmatrix}. \end{aligned}$$

##### Definition 12

Partition $\{X_{1}, \ldots , X_{\ell }\}$ of taxa $[m] \setminus X_{0}$ and partition $\{C_{1},\ldots , C_{\ell }\}$ of characters $C_{*}$ are a *block diagonal decomposition* of $\bar{B}(B,b_*,X_0,C_*)$ if, for every character $c \in C_{i}$, there exists no $t \in X_{j}$ for all j≠i such that $\bar{b}_{t,c} = 1$.

This can be trivially achieved in *O*(*mn*) time by the equivalent problem of, given a bipartite graph’s adjacency matrix, determining its connected components. This equivalency also demonstrates that the block diagonal matrix decomposition of maximum size for any matrix is unique (excluding characters containing all values of 0 in $\bar{B}$, which can be trivially assigned to any $C_{i}$ and have no restricting effect on determining a phylogeny). Thus, we assume we always infer block diagonal matrix decompositions of maximum size. (As a side note, if this size is 1 due to the presence of an all-1 column for example, then no effective decomposition has occurred at all. This implies that $b_*$ cannot actually be a branching point in the first place for the chosen $X_{0}$, $C^+_{0}$, and $C^-_{0}$, so we would not continue forward.) We finally synthesize $B_{0}$, matrix complement $\bar{B}(B,b_*, X_0, C_*)$, and this block diagonal decomposition to formalize a 1-*Dollo matrix decomposition on* *B* *and* $b_{0}$ *by* $X_{0}$, $C^+_{0}$, *and* $C^-_{0}$.

##### Definition 13

Given binary matrix $B \in \{0,1\}^{m \times n}$ and binary vector $b_{0}$, the 1-*Dollo matrix decomposition of* *B* and $b_{0}$ on $X_{0}$, $C^+_{0}$, and $C^-_{0}$ is defined as the set of submatrices $\{B_{0}$, $B_{1}$, ..., $B_{\ell }\}$ such that $B_{0} = B[X_0, C^+_0 \cup C^-_0]$, and $B_{1} = B[X_{1}, C_{1}], \ldots , B_{\ell } = B[X_{\ell }, C_{\ell }]$ are each given by the block diagonal decomposition $\{X_{1}, \ldots , X_{\ell }\}$ and $\{C_{1},\ldots , C_{\ell }\}$ of $\bar{B}(B,b_*,X_0,C_*)$, where $b_{*} = b(b_{0}, C^+_{0},C^-_{0})$ and $C_{*} = [n] \setminus C^-_{0}$, with maximum size ℓ.

Therefore, given binary matrix $B \in \{0,1\}^{m \times n}$ and 1-Dollo phylogeny *T* for *B* rooted at $b_{0}$, we have established a 1-Dollo matrix decomposition of *B* and $b_{0}$ on known sets $X_{0}$, $C^+_{0}$, and $C^-_{0}$ that yields $\{B_{0}$, $B_{1}$, ..., $B_{\ell }\}$. Of course, such a decomposition assumes that the values of $X_{0}$, $C^+_{0}$, and $C^-_{0}$ are indeed correct; that is, that $X_{0}$, $C^+_{0}$, and $C^-_{0}$ are constructed according to the aforementioned recursive characterization of *T* established in Section “inlinkRecursive characterization of rooted 1-Dollo phylogeniesSection:recurseCharT”. Without knowledge of *T*, such values are not trivially known. We use the following definition and lemma to establish a necessary condition to $X_{0}$, $C^+_{0}$, and $C^-_{0}$ being constructed according to this recursive characterization of some *T*, essentially dictating that every character lacking a gain or loss edge in $T_0$ cannot be variable across the taxa in $T_0$.

##### Definition 14

Sets $X_0$, $C^+_0$, $C^-_0$ are *in agreement with* $B \in \{0,1\}^{m \times n}$ and $b_0$ if all characters $c \in [n] \setminus (C^+_{0} \cup C^-_{0})$ are not variable w.r.t submatrix $B[X_{0}, [n]]$ and vector $b_0$.

**Fig. 4:**
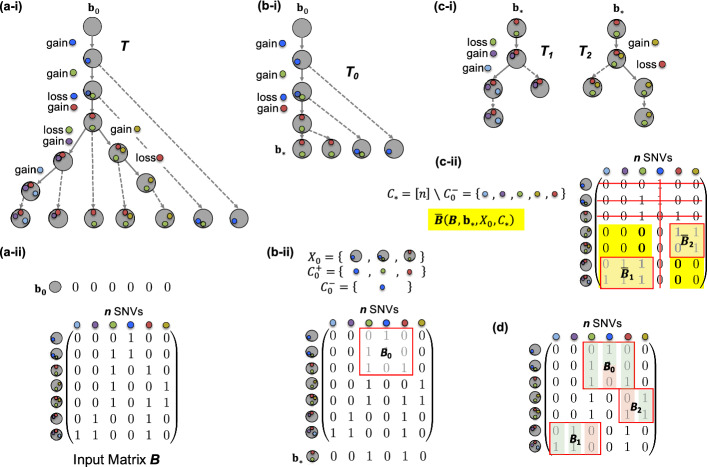
**a-i** An example 1-Dollo phylogeny *T* on **a-ii** matrix *B*, along with the decomposition of *T* into **b-i** 1-Dollo linear phylogeny $T_{0}$ and **c-i** 1-Dollo phylogenies $T_{1}$ and $T_{2}$. This recursive characterization of *T* is allowed by the corresponding 1-Dollo matrix decomposition of *B* by **b-ii** $X_{0}, C^-_0, C^+_0$ into $B_{0}$ and **c-ii** Matrix complement $\bar{B}(B,b_*, X_0, C_*)$ indicated in yellow, with bold entries representing inverted columns, and $B_{1}$ and $B_{2}$. $T_{0}$ is rooted at $b_{0} = 0$ and terminates at $b_{*} = b(b_0, C^+_0, C^-_0)$, and phylogenies $T_{1}$ and $T_{2}$ are rooted at $b_{*}$. Critically, $T_{0}$, $T_{1}$, and $T_{2}$ split the gain and loss edges for each character c∈[n] among themselves such that each character *c* has at most one gain edge and one loss edge among all of $T_{0}$, $T_{1}$, and $T_{2}$. The partition of gain and loss edges are visualized by (d) a color-coding of *B* by green and red, respectively

We now arrive at the main theorem of this section, where we prove that this sole necessary condition, in tandem with the 1-Dollo matrix decomposition of binary matrix *B* and binary vector $b_{0}$ by $X_{0}, C^+_{0}, C^-_{0}$ into $\{B_{0}, B_{1}, \ldots , B_{\ell }\}$, is both necessary and sufficient to recursively characterize all *B* for which there exists a 1-Dollo phylogeny *T* rooted at some $b_{0}$. Intuitively, we prove the forward direction by decomposing an existing 1-Dollo phylogeny *T* on *B* as shown in Section “inlinkRecursive characterization of rooted 1-Dollo phylogeniesSection:recurseCharT”, deriving $X_{0}, C^+_{0}, C^-_{0}$ directly. We then show that each subtree beneath the first-encountered branching point of *T* corresponds to some matrix resulting from the 1-Dollo matrix decomposition of *B* and $b_{0}$ on these derived values. Conversely, we prove the reverse direction by beginning from a 1-Dollo matrix decomposition on some existing $X_{0}, C^+_{0}, C^-_{0}$ and directly constructing *T* from phylogenies on the decomposition’s individual parts. As an aide, we show an example 1-Dollo phylogeny’s recursive decomposition, paralleled by its data matrix’s recursive 1-Dollo decomposition (Fig. 4).

To precisely allow for the composition of phylogenies that each may be on distinct sets of characters, we introduce the notation $b \oplus b_0$ which, given a vector $b \in \{0,1\}^{|C|}$ restricted to characters C⊆[n] and vector $b_0 \in \{0,1\}^n$ on all characters [*n*], re-expands *b* to include all characters in [*n*] by supplementing missing characters with values from $b_0$. For example, given $b = [0, 1]^\top$ on characters $C = \{1, 3\}$ and $b_0 = [0, 1, 0, 1, 1]^\top$ on the full set $\{1,2,3,4,5\}$ of n=5 characters, $b \oplus b_0$ yields $[0, 1, 1, 1, 1]^\top$. Then, the shorthand $T \oplus b_0$ indicates the expansion of all node labels of phylogeny *T*, i.e. $b(v) \oplus b_0$ for all nodes *v* of *T*. We use this notation to state the following theorem.

##### Theorem 2

Given matrix $B \in \{0,1\}^{m \times n}$ and binary vector $b_0$, there exists a 1-Dollo phylogeny *T* for *B* rooted at $b_0$ if and only if there exists some set $X_{0} \subseteq [m]$ of taxa and sets $C^-_{0}, C^+_{0} \subseteq [n]$ of characters subject to the following conditions:

1. Sets $X_0$, $C^+_0$, $C^-_0$ are in agreement with *B* and $b_0$.
2. For the 1-Dollo matrix decomposition of *B* and $b_0$ on $X_0$, $C^+_0$, $C^-_0$ into submatrices $\{B_{0} = B[X_{0}, C_{0}], \ldots , B_{\ell } = B[X_{\ell }, C_{\ell }]\}$, there exists a 1-Dollo linear phylogeny $T_0$ for $B_0$ rooted at $b_0 \oslash C_0$ and terminating on $b_{*} \oslash C_0$ and 1-Dollo phylogenies $T_1,\ldots ,T_\ell$ for $B_1,\ldots ,B_\ell$ rooted at $b(b_0, C^+_0, C^-_0) \oslash C_1,\ldots ,b(b_0, C^+_0, C^-_0)\oslash C_\ell$, respectively.

### Dolphyin

We introduce Dolphyin (DOllo Linear PHYlogeny INference), a combinatorial algorithm that uses the above, recursive, and combinatorial characterization of rooted 1-Dollo phylogenies to solve R1DP altogether. Given any binary matrix *B* and binary vector $b_{0}$, Dolphyin determines a 1-Dollo phylogeny on *B* rooted at $b_{0}$ by exhaustively searching over all possible values of $X_{0}, C^+_{0}, C^-_{0}$ and determining a set of values such that (i) $X_{0}, C^+_{0}, C^-_{0}$ are in agreement with *B* and $b_{0}$ and (ii) given the 1-Dollo matrix decomposition of $X_{0}, C^+_{0}, C^-_{0}$ on *B* and $b_{0}$ into submatrices $\{B_{0}, B_{1}, \ldots , B_{\ell }\}$, there exists a 1-Dollo linear phylogeny $T_{0}$ on $B_0$ rooted at $b_{0}$ terminating on $b_{*} = b(b_{0}, C^+_0, C^-_0)$ and 1-Dollo phylogenies $T_{1}, \ldots , T_{\ell }$ on $B_{1}, \ldots , B_{\ell }$ each rooted at $b_{*}$. This 1-Dollo linear phylogeny $T_{0}$ can be inferred in *O*(*mn*) time, and 1-Dollo phylogenies $T_{1}, \ldots , T_{\ell }$ are then inferred through recursive calls of Dolphyin on matrices $B_{1}, \ldots , B_{\ell }$. Dolphyin then returns the 1-Dollo phylogeny *T* rooted at $b_{0}$ on *B* as the tree formed by the construction on $T_{0}, \ldots , T_{\ell }$ described in Theorem 2. This same Theorem 2 shows that this entire procedure is necessary and sufficient to solve any instance of R1DP. As an additional optimization, Dolphyin preprocesses all matrices by first removing all duplicate columns and rows, as well as trivial columns that contain 0, 1, or *m* 1s.

#### Heuristically determining candidate values of $X_{0}, C^+_{0}, C^-_{0}$

The exhaustive search over $X_{0}, C^+_{0}, C^-_{0}$ as described above is sufficient to determine any existing 1-Dollo phylogeny. However, such a brute-force search over these sets can be computationally intensive, requiring roughly $O(2^m(2^n)^2)=O(2^{m+2n})$ time. Additionally, in practical R1DP instances, the vast majority of possible values of $X_{0}$, $C^+_{0}$, and $C^-_{0}$ in agreement with *B* and $b_{0}$ do not even yield a 1-Dollo linear phylogeny $T_{0}$ on $B_{0}$ rooted at $b_{0}$ and terminating on $b_{*} = b(b_{0},C^+_0, C^-_0)$.

Therefore, we enhance Dolphyin’s performance with a practical heuristic that, prior to the fully exhaustive search, initially restricts the enumerated values of $X_{0}, C^+_{0}, C^-_{0}$ in agreement with *B* and $b_{0}$ to a subset of candidate values where such a $T_{0}$ has already been pre-determined to exist. Specifically, heuristic $\textsc {FindLinearChains}$ constructs a set $\mathcal {T}_{0}$ of precomputed trees $T_{0}$ with corresponding values $X_{0}, C^+_{0}, C^-_{0}$ such that for every element $[b_{0}, T_{0}, X_{0}, C^+_{0}, C^-_{0}] \in \mathcal {T}_{0}$, $T_{0}$ is guaranteed to be a 1-Dollo phylogeny rooted at $b_{0}$ and terminating on $b_{*} = b(b_0,C^+_0, C^-_0)$ on $B_{0} = B[X_{0}, C^+_{0} \cup C^-_{0}]$. Intuitively, $\textsc {FindLinearChains}$ considers every taxon t∈[m] individually and, assuming that taxon *t* is a branching point in *T*, attempts to pack as many taxa as possible into a linear phylogeny beginning with 0 and ending with *t*. Formally, we construct a directed acyclic graph over all taxa with source $b_{0}$ such that an edge between taxa exists if every character observed in $b_{t}$ is not lost along the edge. Then, for every such path in this graph $G_{t}$ beginning with $b_{0}$, we consider all taxa $X_{0}$ in this path and check if such a 1-Dollo linear phylogeny indeed exists on these taxa across all characters. Critically, we record this set of taxa $X_{0}$, along with $T_{0}, C^+_{0}$, and $C^-_{0}$, if and only if a 1-Dollo linear phylogeny exists.

#### Asymptotic runtime of Dolphyin

Even with the above heuristic, the worst-case running time of the initial recursive call in Dolphyin remains $O(2^{m+2n} \cdot mn)$, where the additional factor *mn* corresponds to checking whether the 1-Dollo decomposition of *B* by every possible $X_0,C^+_0,C^-_0$ yields a valid 1-Dollo linear phylogeny.

Now, we will perform a full, worst-case asymptotic runtime analysis of Dolphyin summing over all recursive calls. Let *f*(*m*, *n*) be the worst-case running time of running Dolphyin on an input matrix *B* of size m×n. As a base case, we have that f(1,n)=O(1) and f(m,1)=O(1), since an input with one character or one taxon is trivially solved. Otherwise, Dolphyin examines all $O(2^{m+2n})$ possible combinations of $X_0,C^+_0,C^-_0$ to identify a possible linear phylogeny $T_0$ in *O*(*mn*) time, and for every such combination yielding a viable $T_0$ recurses on block-diagonal decomposed matrices $B_{1}, \ldots , B_{\ell }$ as described above. In the worst case scenario, all possible combinations of $X_0,C^+_0,C^-_0$ yield a linear phylogeny $T_0$ and thus necessitate recursion. Furthermore, a block decomposition results in $\ell \ge 2$ non-empty blocks, so in the worst case scenario, the matrices $B_{1}, \ldots , B_{\ell }$ are also as few, large, and size-imbalanced as possible (when ℓ=2, $B_{1}$ has a single taxon and character, and $B_{2}$ has at most m-1 taxa and n-1 characters). As a result, we can write the worst-case analytical runtime of Dolphyin as:

6 $$\begin{aligned} f(m, n)&= {\left\{ \begin{array}{ll} O(2^{m+2n} \cdot mn + 2^{m+2n} \cdot f(m-1,n-1)), &  {m \ge 2 \wedge n \ge 2,}\\ O(1), &  {m=1 \vee n=1,} \end{array}\right. } \end{aligned}$$

7 $$\begin{aligned}&= \sum _{i=0}^{m}\left[ \prod _{j=0}^i O(2^{(m-j)+2(n-j)})\right] mn. \end{aligned}$$

Intuitively, $[\prod _{j=0}^i O(2^{(m-j)+2(n-j)})]mn$ is the amount of non-recursive work done in any layer i∈[m] of recursion, and we simply sum over all layers. We derive this layer-specific term itself from the fact that the number of total recursive calls to Dolphyin at any layer *i* is the same as the previous layer’s number of calls times $2^{(m-i)+2(n-i)}$, and the amount of actual non-recursive work done in any such call individually is O((m-i)(n-i))=O(mn). So, using this pattern of exponentially-increasing calls over layers and the upper bound of *O*(*mn*) for work within any call, the last layer of recursion in our analysis clearly dominates runtime. The greatest possible number of layers of recursion is min(m,n), so we continue with

8 $$\begin{aligned} f(m, n)&= O(\left[ \prod _{j=0}^{\min (m, n)} 2^{(m-j)+2(n-j)}\right] mn) \end{aligned}$$

9 $$\begin{aligned}&= O(\left[ 2^{\sum _{j = 0}^{m}j+2\sum _{j = 0}^{n}j}\right] mn) \end{aligned}$$

10 $$\begin{aligned}&= O(\left[ 2^{\frac{m(m-1) + 2n(n-1)}{2}}\right] mn) \end{aligned}$$

11 $$\begin{aligned}&= O(\sqrt{2}^{[m(m-1) + 2n(n-1)]} mn) \end{aligned}$$

So our final worst-case asymptotic runtime for Dolphyin can be characterized as $O(\sqrt{2}^{[m(m-1) + 2n(n-1)]} mn)$, which is super-exponential.

We additionally provide a separate, but very similar runtime analysis under the more optimistic assumption that 1-Dollo matrix decompositions yield submatrices that are roughly balanced in size (but still as large and few as possible with ℓ=2). Specifically, under this assumption both ℓ=2 recursive calls would have equal runtimes of $f(\frac{m}{2}, \frac{n}{2})$ each, so we now begin in the recursive case from $f(m, n) = O(2^{m+2n} \cdot mn + 2^{m+2n} \cdot 2f(\frac{m}{2}, \frac{n}{2}))$.

12 $$\begin{aligned} f(m, n)&= {\left\{ \begin{array}{ll} O(2^{m+2n} \cdot mn + 2^{m+2n} \cdot 2f(\frac{m}{2}, \frac{n}{2})), &  {m \ge 2 \wedge n \ge 2,}\\ O(1), &  {m=1 \vee n=1,} \end{array}\right. } \end{aligned}$$

13 $$\begin{aligned}&= \sum _{i=0}^{m}\left[ \prod _{j=0}^i O(2^i \cdot 2^{(\frac{m}{2^j})+2(\frac{n}{2^j})})\right] mn. \end{aligned}$$

The final layer of recursion thus dominates again by the same argument as before, and there are now at most $i = \min {(\log m, \log n)}$ layers, so

14 $$\begin{aligned} f(m, n)&= O(\left[ \prod _{j=0}^{\min {(\log m, \log n)}} 2^{\min {(\log m, \log n)}} \cdot 2^{(\frac{m}{2^j})+2(\frac{n}{2^j})} \right] mn) \end{aligned}$$

15 $$\begin{aligned}&= O(\min {(m,n)} \left[ \prod _{j=0}^{\min {(\log m, \log n)}} 2^{(\frac{m}{2^j})+2(\frac{n}{2^j})} \right] mn ) \end{aligned}$$

16 $$\begin{aligned}&= O(\min {(m,n)} \left[ 2^{\sum _{j=0}^{\min {(\log m, \log n)}}(\frac{m}{2^j})+ \sum _{j=0}^{\min {(\log m, \log n)}}2(\frac{n}{2^j})} \right] mn) \end{aligned}$$

17 $$\begin{aligned}&= O(\min {(m,n)} \left[ 2^{2m+2(2n)}\right] mn) \end{aligned}$$

18 $$\begin{aligned}&= O(\min {(m,n)} 4^{[m+2n]} mn) \end{aligned}$$

So, under this assumption of balanced recursive calls, Dolphyin yields an exponential, but noticeably lower, asymptotic runtime of $O(\min {(m,n)} 4^{[m+2n]} mn)$. Together, these runtimes of $O(\sqrt{2}^{[m(m-1) + 2n(n-1)]} mn)$ in the absolute worst case and $O(\min {(m,n)} 4^{[m+2n]} mn)$ in the case of minimally-decomposed, but balanced, recursive calls characterize Dolphyin’s theoretical performance.

#### Adapting Dolphyin to false negatives in data

When examining simulated datasets with false negative errors or real data, we modified Dolphyin to probabilistically employ error correction. Specifically, in every recursive call, Dolphyin randomly considers p=0.25 pairs of taxa with replacement and, if the normalized Hamming distance over characters between both taxa is less than or equal to some value $0 \le e \le 1$, alter any character seen in one taxon to be present in both taxa. For each pair of taxa, one taxon was selected with uniform probability and the other was selected with probability inversely proportional to each row’s prevalence in the dataset. To perform analysis on any given dataset with errors, we initially let e=0, which equates to no error correction, and iteratively increased *e* by 0.2 until Dolphyin returned a solution within a 10 second time limit.

## Results

We implemented Dolphyin in C++11 and ran all analyses, including the comparison to SPhyR, on an Apple 2.3 GHz 8-Core Intel Core i9 Macbook Pro. Dolphyin is available at: https://github.com/elkebir-group/Dolphyin with commit hash fbf400f, tag v1.0.0 used for the experiments in this paper.

### Results on simulated data

**Fig. 5:**
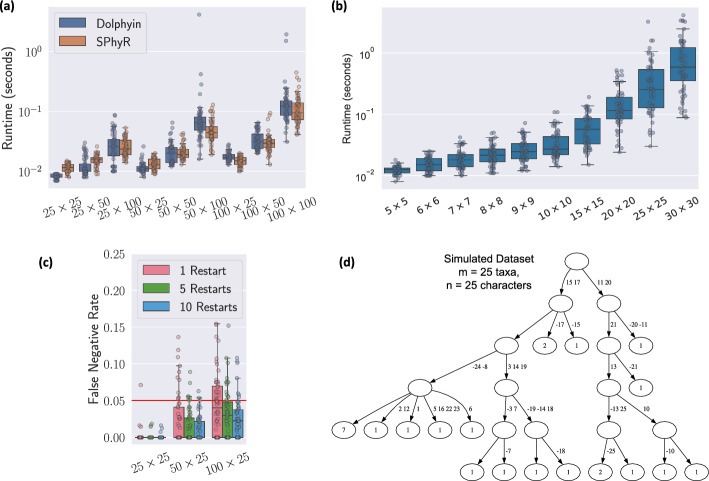
**a** Dolphyin’s runtime on simulated, errorless datasets in comparison to that of ILP-based method SPhyR [12]. **b** Dolphyin’s runtime to determine that no possible errorless 1-Dollo phylogeny exists on simulated, errorless datasets guaranteed to have no solutions. **c** The false negative error rate of data inferred by Dolphyin on simulated datasets randomly augmented with false negatives at a ground truth rate (red line) of 0.05. **d** An example phylogeny returned by Dolphyin on a dataset with false negatives and m=n=25 taxa and characters. Nodes are annotated by the number of taxa present; edges are annotated by characters numbered 1 to 25. Loss events are indicated by a minus sign

We first used Dolphyin to analyze 540 simulated matrices of errorless, single-cell sequencing data with 1-Dollo ground truth phylogenies. These datasets had either $m \in \{25, 50, 100\}$ cells and $n \in \{25, 50, 100\}$ SNVs, with 60 datasets per combination of *m* and *n*, and were previously used to benchmark the ILP-based *k*-Dollo solver SPhyR (Fig. 5a). Data was generated using ms [29], and full details of data generation and the data itself are accessible from SPhyR’s initial publication [12]. On all 540 examined instances, Dolphyin found and returned valid errorless 1-Dollo phylogenies. We found that Dolphyin remained competitive with SPhyR in the majority of test cases across all input sizes, with identical median running times of 0.019 seconds for both methods across all instances. While Dolphyin slightly outperformed SPhyR on the smaller input sizes (for 25×25 instances: 0.008 seconds for Dolphyin and 0.0115 seconds for SPhyR), SPhyR slightly outperformed Dolphyin on larger input sizes (for 100×100 instances: 0.122 seconds for Dolphyin and 0.0945 seconds for SPhyR).

Next, we examined Dolphyin’s ability to analyze datasets that were pre-determined to have no satisfying errorless 1-Dollo phylogenies. To construct simulated data that were comparable to the above datasets but guaranteed to have no errorless solution, we augmented the 60 simulated datasets described above of size m=50, n=50 by replacing the intersection of each dataset’s first 4 rows and first 4 columns with the following matrix:

19 $$\begin{aligned} \begin{pmatrix} 0 \hspace{5pt}&  0 \hspace{5pt} &  1 \hspace{5pt}&  1\\ 0 \hspace{5pt}&  1 \hspace{5pt}&  0 \hspace{5pt}&  1\\ 1 \hspace{5pt}&  0 \hspace{5pt}&  1 \hspace{5pt}&  0\\ 1 \hspace{5pt} &  1 \hspace{5pt}&  0 \hspace{5pt}&  0 \end{pmatrix} \end{aligned}$$

This is the smallest matrix known to have no valid 1-Dollo phylogeny on its four taxa and characters [30], so any dataset that contains this submatrix also will necessarily have no 1-Dollo phylogeny regardless of total size. We used Dolphyin to analyze each of these 60 augmented matrices when restricted to the first $m = n \in \{5, 6, 7, 8, 9, 10, 15, 20, 25, 30\}$ taxa and characters, effectively analyzing a total of 600 matrices across a range of sizes that all theoretically are guaranteed to have no solution. Dolphyin, despite not explicitly searching for this 4×4 matrix, correctly determined that there existed no satisfying errorless 1-Dollo phylogenies for any of these 600 matrices. The runtime required to determine this on each matrix instance increases roughly exponentially with matrix size (Fig. 5b) and is, on average, greater than the time required to find the existence of a solution when one does exist (Fig. 5a). Specifically, Dolphyin has a median runtime of 0.013 seconds on input size m=n=5 and a median runtime of 0.589 seconds on size m=n=30. The range of these runtimes over instances increases greatly with size also, showing minimum and maximum runtimes of 0.008 and 0.018 seconds on m=n=5 respectively (range of 0.010 seconds) and minimum and maximum runtimes of 0.088 and 4.095 seconds on m=n=30 respectively (range of 4.007 seconds).

Finally, to assess Dolphyin’s ability to infer error-allowing—and thus more practical—1-Dollo phylogenies on datasets containing false negatives, we augmented the 180 simulated datasets of sizes $m \in \{25, 50, 100\}$, n=25 by flipping each 1 in these datasets to a 0 with a false-negative error rate of 0.05. We chose to augment matrices of these sizes because the number of characters n=25 was most comparable to that of the real AML data we examined afterwards. Similarly, we chose an error rate of 0.05 in simulations because of its similarity to the estimated median false negative rate of 0.058 predicted under the Mission Bio Tapestri sequencing technology producing real AML data (allelic dropout rate of 5.8%) [20]. Since Dolphyin’s false negative error correction is inherently probabilistic, we analyzed each dataset with 1, 5, or 10 restarts of Dolphyin. We report the lowest inferred false negative rate of all restarts (Fig. 5c), which corresponds to the 1-Dollo phylogeny best fitting the data. In the median case, Dolphyin inferred phylogenies with a false negative rate at or below the ground truth rate used to generate the data (m=100; 1 restart: median rate of 0.0399, 5 restarts: median rate of 0.0301, 10 restarts: median rate of 0.0225). Predictably, we found that increasing the number of restarts decreased the error rate of the best 1-Dollo phylogeny inferred. As an example, we provide a 1-Dollo phylogeny returned by Dolphyin in the analysis of a dataset with m=25 taxa and n=25 characters (Fig. 5d). While Dolphyin is based on the characterization of 1-Dollo linear phylogenies, it clearly determines 1-Dollo phylogenies with branching.

**Fig. 6:**
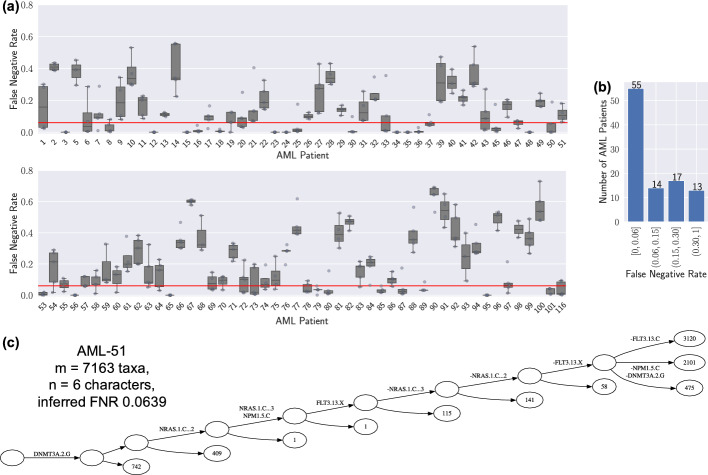
**a** The inferred false negative rates for all 99 examined AML datasets over 5 restarts per dataset, relative to the false negative rate expected of the sequencing technology (0.058, red line) [20]. **b** Among all restarts, slightly over half (55) of the AML datasets yielded phylogenies with a false negative rate lower than 0.06. **c** The phylogeny returned by Dolphyin on real dataset AML 51 [20] with m=7163 taxa and n=6 characters. Nodes are annotated by the number of taxa present; edges are annotated with characters, or SNVs. Loss edges are indicated by a minus sign

### Results on AML data

Having used Dolphyin to analyze simulated datasets both with and without false-negative sequencing errors, we then used Dolphyin to analyze 99 real sets of AML single-cell sequencing data [20] processed in a previous work [23] with 5 restarts per dataset. Prior to analysis, we removed all cells with unsequenced or unknown characters, yielding a mean of m=5460 taxa, or cells, and n=4.42 characters per dataset.

We show the error rates achieved on each dataset across all 5 restarts (Fig. 6a), demonstrating a moderate level of consistency in inferred error rates between restarts (standard deviation between restarts, averaged over datasets of 0.0634). Taking the minimum over all 5 restarts, Dolphyin inferred phylogenies on the majority (55) of datasets with an estimated false negative error rate at or below 0.06 (Fig. 6b), approximately matching the Mission Bio Tapestri sequencing technology’s median allelic dropout rate of 5.8% [20]. We speculate that for those datasets on which error rates much greater than 0.06 were inferred, there are three possibilities. Firstly, false positives in the real data, while less likely than false negatives (estimated false positive rate of 1% in initial work [20]), may be preventing Dolphyin from finding phylogenies with realistic error rates. Secondly and thirdly, more than k=1 losses may be necessary to realistically explain these datasets, or simply more restarts of Dolphyin may be required to locate a realistic solution.

Finally, as a precise example of the 1-Dollo phylogenies inferred by Dolphyin, we supply the mutation-annotated phylogeny Dolphyin inferred on AML dataset 51 with size m=7163, n=6 with a false negative error rate of 0.0639 (Fig. 6c). Dolphyin inferred that AML dataset 51 had a near-linear 1-Dollo phylogeny with an internal node of outdegree 3 furthest from the root.

## Conclusion

This work examined the problem of inferring a 1-Dollo, or persistent phylogeny on single-cell sequencing DNA data for SNVs. We first examined the subcase in which our 1-Dollo phylogeny must be linear, and we proved an equivalence between whether a binary data matrix *B* admits a 1-Dollo linear phylogeny and whether *B* has the known consecutive ones property, which can be verified in polynomial time [24]. We also developed polynomial-time algorithms for natural extensions of the 1-Dollo linear subcase, including the cases where we restrict the 1-Dollo phylogeny to be rooted at and/or terminate at some states. Using the linear subcase, we recursively characterized all binary matrices that admit a 1-Dollo phylogeny with a series of conditions that are provably necessary and sufficient. Unfortunately, determining whether a matrix *B* is 1-Dollo using this characterization takes exponential time, leaving the hardness of the 1-Dollo phylogeny problem open. In addition, we showed that the problem of minimizing false negatives in data as to admit a 1-Dollo linear phylogeny is NP-hard.

We used these theoretical results to develop Dolphyin, a combinatorial algorithm that infers 1-Dollo phylogenies from sequencing data. Dolphyin directly leverages our above characterization of matrices admitting 1-Dollo phylogenies by identifying 1-Dollo linear phylogenies on subsets of taxa and recursing on remaining taxa and characters. Dolphyin also incorporates probabilistic error correction and thus can also be applied to data with false negative sequencing errors. We performed an analysis of Dolphyin’s full asymptotic runtime and concluded that Dolphyin is, in its worst case, theoretically exponential in both the number of taxa and characters. In practice, we used Dolphyin to first analyze errorless, simulated datasets and showed that Dolphyin is runtime competitive with SPhyR [12], a previous ILP-based approach for inferring *k*-Dollo phylogenies. On simulated datasets guaranteed to have no solution, Dolphyin consistently identifies that no errorless solution exists. Dolphyin’s exponential runtime to prove the lack of an errorless solution is the best practical reflection of our analytically-derived runtime, since the analytically-derived runtime also assumes that no solution is found early and thus that a complete, exhaustive search of exponential possibilities is required.

We then applied Dolphyin to simulated datasets with false negative errors and demonstrated that Dolphyin, in the median case, infers 1-Dollo phylogenies with an inferred error rate at or below the ground truth rate. We finally applied Dolphyin to 99 real acute myeloid leukemia datasets [20] and found that Dolphyin infers 1-Dollo phylogenies on the majority of these datasets with an error rate at or below the previously, experimentally-estimated false negative error rate specific to the sequencing technology producing these datasets.

In future work, we may consider more advanced error correction schemes (both building on the allowance of false negatives, and extensions to false positives) for more widely applying Dolphyin to existing datasets. Our exploration of Dolphyin’s practical, exponential runtime of matrices known to have no solution due to minimal changes demonstrates a scalability limit to the number of taxa or characters when the data does not obey the 1-Dollo phylogeny model, so better heuristics that do not sacrifice correctness will be critical for scaling analysis to datasets with thousands of cells or characters. We may also attempt to extend a similar, combinatorial and recursive framework to the *k*-Dollo model of evolution for k>1, or models of evolution with more than one gain. However, we note that even determining a specifically linear and errorless phylogeny with two gains or losses, per character, is NP-hard [28]. Additionally, while we may consider problem extensions such as determining a maximal number of taxa or characters admitting 1-Dollo phylogenies in data, we note that the equivalence of 1DLP to the consecutive ones property makes several natural formulations for identifying maximal data subsets NP-hard in even the linear and errorless cases [31]. Another natural problem extension with experimental applications worth discussing is determining whether a 1-Dollo phylogeny is feasible or not on single-cell datasets with ambiguous entries; that is, datasets where the state of some characters for some cells is unknown. This is highly applicable when sequencing coverage is too low at some positions to reliably call a cell’s genotype as 0 or 1. Unlike the error-allowing ME1DLP problem—where a solution can flip any entry at a per-flip cost—here ambiguous entries can be assigned either state for free, but only those entries are free to vary; fixed 0s and 1s remain fixed. The equivalent problem regarding perfect phylogenies can be conceptualized by the exact requirement that ambiguous entries must not induce a specific 3 by 2 submatrix [32]. While there currently exists no comprehensive characterization of such forbidden submatrices for 1-Dollo phylogenies [30], this remains a promising approach.

In summary, our work adds to the theoretical body of knowledge on the 1-Dollo, or persistent phylogeny, model of evolution and provides a practical algorithm for inferring phylogenies that leverages these theoretical results. We hope that this combinatorial approach will aid advances in determining the complexity of 1-Dollo problems and their variants.

## Data Availability

No datasets were generated or analysed during the current study.

## Appendix

### Lemma 1

For any 1-Dollo phylogeny *T* for a matrix *B*, there exists a unique compact 1-Dollo phylogeny $T^{\prime }$, obtainable from *T*, for *B*.

### Proof of Lemma 1

Let *T* be a 1-Dollo phylogeny for matrix *B*. First, we contract all internal edges (*u*, *v*) of *T* such that $$b_T(u) = b_T(v)$$. Then, let *U* be the set of resulting internal, non-root nodes *u* of *T* that do not correspond to observed taxa nor are branching points (Definition 3). If *U* is empty, then we have already obtained the unique compact 1-Dollo phylogeny for matrix *B* obtained from *T*. If *U* is non-empty, consider any node u∈U and let $u^{\prime }$ be the parent of *u* (which exists as u≠r). Since *u* is an internal node, we have that *u* does not correspond to a taxon and that *u* is not a branching point. Therefore, *u* has exactly one child *v*. We obtain $T^{\prime }$ by removing the edges $\left(u^{\prime },u\right)$ and (*u*, *v*), removing the node *u*, and inserting the new edge $\left(u^{\prime },v\right)$. Clearly, $T^{\prime }$ remains a 1-Dollo phylogeny for *B*. Removing all nodes in *U* (in any order) yields the unique compact 1-Dollo phylogeny for matrix *B* obtained from *T*. □

### Theorem 1

There exists a 1-Dollo linear phylogeny *T* for *B* if and only if *B* is C1P.

### Proof of Theorem 1

(⇒) Let *T* be a 1-Dollo linear phylogeny for matrix $$B \in \{0,1\}^{m \times n}$$. By Definition 1, we have that *T* has exactly *m* leaves $$v_1,\ldots ,v_m$$ with incoming edges $$(u_1,v_1),\ldots ,(u_m,v_m)$$ such that $$b_1 = b(u_1) = b(v_1),\ldots ,b_m = b(u_m) = b(v_m)$$. Moreover, removal of these *m* leaves results in a linear, chain graph $T^{\prime }$, containing nodes $$u_1,\ldots ,u_m$$ and root *r*. Performing a pre-order traversal of $T^{\prime }$ starting from *r* yields an ordering $$u_{\pi (1)},\ldots ,u_{\pi (m)}$$ of the nodes $$u_1,\ldots ,u_m$$ and therefore the *m* taxa of *B*. Note that by Definition 1 each character is gained at most once, and if gained, lost at most once. Therefore, for each character c∈[n], all taxa t∈[m] such that $$b_{t,c}=1$$ will appear consecutively in π. Hence, *B* is C1P as certified by permutation π.

(⇐) Let $$B \in \{0,1\}^{m \times n}$$ be C1P. As such, there exists a permutation $$\pi : [m] \rightarrow [m]$$ such that for each column *c* there exists at most one row *t* such that $$b_{\pi (t-1), c} = 0$$ and $$b_{\pi (t), c} = 1$$ and at most one row $t^{\prime }$ such that $$b_{\pi (t'-1), c} = 1$$ and $$b_{\pi (t'), c} = 0$$. Let *T* be the tree obtained from T(B,π) following Definition 5. We claim that *T* is a 1-Dollo phylogeny for *B* in line with Definition 1. Clearly, each node *v* of *T* is labeled by a binary vector $$b(v) \in \{0,1\}^n$$ (condition (i)), the root $$r = u_0$$ is labeled by b(r)=0 (condition (ii)) and each taxon t∈[m] corresponds to a unique leaf $$v_t = \sigma (t)$$ with parent $$u_t$$ such that $$b(v_t)=b(u_t)=b_{\pi (t)}$$ (condition (iii)). It remains to show that there is at most one gain edge and at most one loss edge (condition (iv)) for each character c∈[n]. However, since the permutation π details at most one interval of consecutive taxa for each character *c*, it follows that taxa in *T* gain *c* through at most one gain edge and lose *c* through at most one subsequent loss edge. Condition (v) is similarly satisfied with the further specification that there is no gain edge for any character *c* where $$b_{0,c} = 1$$. Hence, *T* is a 1-Dollo phylogeny for *B*. □

### Lemma 2

There exists a 1-Dollo linear phylogeny *T* for *B* rooted at $$b_0$$ if and only if there exists a 1-Dollo linear phylogeny $T^{\prime }$ for $$B'(B,b_0)$$.

### Proof of Lemma 2

(⇒) Let *T* be a 1-Dollo linear phylogeny for matrix *B* rooted at $$b_0$$. Moreover let $$B' = B'(B,b_0)$$ be the (m+1)×(n+1) matrix obtained from *B* and $$b_0$$ following Definition 6. Given *T*, we will construct a 1-Dollo linear phylogeny $T^{\prime }$ for $B^{\prime }$. Specifically, we construct $T^{\prime }$ from *T* by re-rooting on an appended node $r^{\prime }$ labeled by the *n*-dimensional vector $b(r^{\prime })=0$ and adding the edge $\left(r^{\prime },r\right)$. We additionally add leaf $$v_{0}$$ labeled by $$b_0$$ and include the edge $$(r,v_0)$$. Then, we extend the *n*-dimensional binary vector $$b(v) = [b_{v,1},\ldots ,b_{v,n}]^\top $$ for each node *v* of $T^{\prime }$ with an additional entry $$b_{v,n+1}$$ defined as

A1 $$\begin{aligned} b_{v,n+1} = {\left\{ \begin{array}{ll} 0, &  \text{ if } v \in \{r,r'\},\\ 1, &  \text{ if } v \not \in \{r,r'\}. \end{array}\right. } \end{aligned}$$

We claim that $T^{\prime }$ is a 1-Dollo linear phylogeny for $B^{\prime }$. Clearly, $T^{\prime }$ is node-labeled and rooted at 0. By virtue of the fact that *T* is a 1-Dollo linear phylogeny for matrix $$B \in \{0,1\}^{m \times n}$$ rooted at $$b_0$$, every character c∈[n] has at most one gain edge and one loss edge in $T^{\prime }$, and every original taxon t∈[m] is present in $T^{\prime }$. Additionally, character c=n+1 has at most one gain edge outgoing from *r* and no loss edges, and the new taxon m+1 corresponds to the leaf $$\sigma (m+1) = v_r$$. Finally, the construction of $T^{\prime }$ from *T* retains the linearity of *T*.

(⇐) Let $T^{\prime }$ be a 1-Dollo linear phylogeny for $B^{\prime }$ such that $T^{\prime }$ is compact. Following Theorem 1, let $$\pi : [m] \rightarrow [m]$$ be the permutation such that $T\left(B,\pi \right)=T^{\prime }$.

Since $T^{\prime }$ is linear, the root node $r^{\prime }$ of $T^{\prime }$ must have at most one non-leaf child. If no such node exists then *B* trivially has m=0 taxa and the tree $T^{\prime }$ consisting of a single node labeled by $$b_0$$ is a 1-Dollo linear phylogeny for *B* rooted at $$b_0$$.

We now focus on the case where *B* has m>0 taxa. Let *r* be the non-leaf child of the root node $r^{\prime }$ of $T^{\prime }$. Since $$b'_{t,n + 1} = 1$$ for all t∈[m] and $$b'_{m + 1,n + 1} = 0$$, it must be the case that either π(m+1)=1 or π(m+1)=m+1. To see why observe that 1<π(m+1)<m+1 would imply that character n+1 would have two gain edges within $T^{\prime }$, violating the definition of a 1-Dollo linear phylogeny. We may assume without loss of generality on $T^{\prime }$ that π(m+1)=1, since if π(m+1)=m+1, then for the permutation $$\pi ^*$$ such that $$\pi ^*$$ reverses π, tree $$T^* = T(B, \pi ^*)$$ would be a 1-Dollo linear phylogeny for *B* such that $$\pi ^*(m + 1) = 1$$. Therefore, since π(m+1)=1, it must be the case that *r* is labeled by $$b_0$$.

Given $T^{\prime }$, we will now construct a 1-Dollo linear phylogeny *T* for matrix *B* rooted at $$b_0$$. Let $$v_r$$ be the leaf of $T^{\prime }$ whose parent is *r*. Specifically, we define *T* as the subtree of $T^{\prime }$ rooted at *r* that excludes the leaf $$v_{r}$$. We relabel each node *v* of *T* omitting the (n+1)th entry of its original label $$b_{T'}(v)$$.

Since *T* contains all gain and loss edges present in $T^{\prime }$ precisely excluding edges $\left(r^{\prime },r\right)$ and $$(r,v_{r})$$, contains all taxa in $T^{\prime }$ precisely excluding leaf taxon $$v_{r}$$, and has root label $$b_{0}$$, we have that *T* is a 1-Dollo linear phylogeny *T* for *B* rooted at $$b_0$$. □

### Lemma 3

There exists a 1-Dollo linear phylogeny *T* for *B* rooted at $$b_0$$ and terminating at $$b_*$$ if and only if there exists a 1-Dollo linear phylogeny $T^{\prime }$ for $$B'(B,b_0,b_*)$$.

### Proof of Lemma 3

(⇒) Let *T* be a 1-Dollo linear phylogeny for matrix *B* rooted at $$b_0$$ and terminating at $$b_*$$. Moreover let $$B' = B'(B,b_0,b_*)$$ be the (m+2)×(n+2) matrix obtained from *B*, $$b_0$$ and $$b_*$$ following Definition 8. Given *T*, we will construct a 1-Dollo linear phylogeny $T^{\prime }$ for $B^{\prime }$. Let *r* be the root of *T*. Since *T* is linear, removal of the *m* leaves corresponding to the *m* taxa yields a directed chain graph. Let *s* be the sink node of this graph. Specifically, we construct $T^{\prime }$ from *T* by re-rooting on an appended node $r^{\prime }$ labeled by the *n*-dimensional vector $b(r^{\prime })=0$ and adding the edge $\left(r^{\prime },r\right)$. We additionally add leaf $$v_{0}$$ labeled by $$b_0$$ and include the edge $$(r,v_0)$$. Next, we add a leaf $s^{\prime }$ labeled by $$b_*$$ and include the edge $\left(s,s^{\prime }\right)$. Then, we extend the *n*-dimensional binary vector $$b(v) = [b_{v,1},\ldots ,b_{v,n}]^\top $$ for each node *v* of $T^{\prime }$ with an two additional entries $$b_{v,n+1}$$ and $$b_{v,n+2}$$ defined as

A2 $$\begin{aligned} b_{v,n+1} = {\left\{ \begin{array}{ll} 0, &  \text{ if } v \in \{r,r'\},\\ 1, &  \text{ if } v \not \in \{r,r'\}, \end{array}\right. } \quad \text{ and }\quad b_{v,n+2} = {\left\{ \begin{array}{ll} 0, &  \text{ if } v \in \{r,s,s'\},\\ 1, &  \text{ if } v \not \in \{r,s,s'\} \end{array}\right. } \end{aligned}$$

We claim that $T^{\prime }$ is a 1-Dollo linear phylogeny for $B^{\prime }$. Clearly, $T^{\prime }$ is node-labeled and rooted at 0. By virtue of the fact that *T* is a 1-Dollo linear phylogeny for matrix $$B \in \{0,1\}^{m \times n}$$ rooted at $$b_0$$, every character c∈[n] has at most one gain edge and one loss edge in $T^{\prime }$, and every original taxon t∈[m] is present in $T^{\prime }$. Character c=n+1 has at most one gain edge outgoing from *r* and no loss edges, and the new taxon m+1 corresponds to the leaf $$\sigma (m+1) = v_r$$. Character c=n+2 has at most one gain edge $\left(r^{\prime },r\right)$ and at most one loss edge incoming to *s*, and the new taxon m+2 corresponds to the leaf $$\sigma (m+2) = v_s$$. Finally, the construction of $T^{\prime }$ from *T* retains the linearity of *T*.

(⇐) Let $T^{\prime }$ be a 1-Dollo linear phylogeny for $B^{\prime }$ such that $T^{\prime }$ is compact. Following Theorem 1, let $$\pi : [m] \rightarrow [m]$$ be the permutation such that $T\left(B,\pi \right)=T^{\prime }$. Since $T^{\prime }$ is linear, the root node $r^{\prime }$ of $T^{\prime }$ must have at most one non-leaf child. If no such node exists then *B* trivially has m=0 taxa and the tree $T^{\prime }$ consisting of nodes $$\{r, s\}$$ with edge (*r*, *s*) such that *r* is labeled by $$b_0$$ and *s* is labeled by $$b_*$$ is a 1-Dollo linear phylogeny for *B* rooted at $$b_0$$ terminating at $$b_*$$.

We now focus on the case where *B* has m>0 taxa. Let *r* be the non-leaf child of the root node $r^{\prime }$ of $T^{\prime }$, and let *s* be the sink node of the tree constructed by removing all leaves from $T^{\prime }$. Since $$b'_{t,n + 1} = 1$$ for all $$t \in \{1,2,\ldots ,m,m+2\}$$ and $$b'_{m + 1,n + 1} = 0$$, it must be the case that either π(m+1)=1 or π(m+1)=m+2. To see why observe that 1<π(m+1)<m+2 would imply that character n+1 would have two gain edges within $T^{\prime }$, violating the definition of a 1-Dollo linear phylogeny. By the same logic, since $$b'_{t,n + 2} = 1$$ for all $$t \in \{1,2,\ldots ,m,m+1\}$$ and $$b'_{m + 2,n + 2} = 0$$, it must be the case that either π(m+2)=1 or π(m+2)=m+2. However, it is clear that $$\pi (m + 1) \ne \pi (m + 2)$$. Therefore, it must be the case that either (i) π(m+1)=1 and π(m+2)=m+2, or (ii) π(m+1)=m+2 and π(m+2)=1.

We may assume without loss of generality on $T^{\prime }$ that this first case holds, namely, that π(m+1)=1 and π(m+2)=m+2. If π(m+1)=m+2 and π(m+2)=1, then for the permutation $$\pi ^*$$ such that $$\pi ^*$$ reverses π, tree $$T^* = T(B, \pi ^*)$$ would be a 1-Dollo linear phylogeny for *B* such that $$\pi ^*(m + 1) = 1$$. Since π(m+1)=1 and π(m+2)=m+2, it must be the case that *r* is labeled by $$b_0$$ and *s* is labeled by $$b_*$$.

Given $T^{\prime }$, we will now construct a 1-Dollo linear phylogeny *T* for matrix *B* rooted at $$b_0$$. Let $$v_r$$ be the leaf of $T^{\prime }$ whose parent is *r*, and let $s^{\prime }$ be the leaf of $T^{\prime }$ whose parent is *s*. Specifically, we define *T* as the subtree of $T^{\prime }$ rooted at *r* that excludes the leaf $$v_{r}$$ and leaf $s^{\prime }$. We relabel each node *v* of *T* omitting the (n+1)th and (n+2)th entries of its original label $$b_{T'}(v)$$. □

### Lemma 4

For a given binary vector $b_0 \in \{0,1\}^n$ and 1-Dollo phylogeny *T* for matrix $B \in \{0,1\}^{m \times n}$, let $T_0$ be the subtree of *T* obtained by traversing from the node $v_0$ labeled by $b_0$ to a first branching point $v_*$ with label $b_*$, and let $T_1,\ldots ,T_\ell$ be the subtrees of *T* obtained by traversing along each of the $\ell \ge 2$ outgoing edges from $v_*$. Let $C^+_i$, $C^-_i$ and $X_i$ be the gained characters, lost characters and observed taxa, respectively, in tree $T_i$ where $i \in \{0,\ldots ,\ell \}$. Let $C_i = C^-_i \cup C^+_i$ for all $i \in \{0,\ldots ,\ell \}$. Then, the following conditions hold.

1. Sets $C^+_{0},\ldots ,C^+_{\ell }$ are pairwise disjoint, sets $C^-_{0},\ldots ,C^-_{\ell }$ are pairwise disjoint, and sets $C_{1},\ldots ,C_{\ell }$ are pairwise disjoint.
2. Sets $X_0,\ldots ,X_\ell$ are pairwise disjoint, and $X_{0} \cup \ldots \cup X_\ell$ is the set of all taxa observed in the subtree of *T* rooted at $v_0$.
3. $$C^+_i \subseteq [n] \setminus C^-_0$$ for all $$i \in [\ell ]$$.
4. Tree $T_0 \oslash C_0$ is a 1-Dollo linear phylogeny for submatrix $B_{0} = B[X_0,C_0]$ rooted at $b_{0} \oslash C_0$ terminating at $b_* \oslash C_0$.
5. For each $i \in [\ell ]$, tree $T_i \oslash C_i$ is a 1-Dollo phylogeny for submatrix $B_{i} = B[X_i,C_i]$ rooted at $b_{*} \oslash C_i$.

### Proof of Lemma 4

We will prove the above conditions in the order they were proposed.

1. Since *T* itself is a 1-Dollo phylogeny and characters can thus only be gained and lost once throughout all of *T*, it holds that $C^+_{1},\ldots ,C^+_{\ell }$ and $C^-_{1},\ldots ,C^-_{\ell }$ are each pairwise disjoint. Additionally, sets $C^+_{i}$ and $C^-_{j}$ for all distinct $i, j \in [\ell ]$ must be disjoint, since no character *c* can be gained in one subtree and lost in another subtree rooted at the same branching point $v_*$. Hence, $C_{1},\ldots ,C_{\ell }$ must additionally be pairwise disjoint.
2. Sets $X_0,\ldots ,X_\ell$ are pairwise disjoint since, by definition, each taxon t∈[m] is observed as a leaf exactly once in *T*, and these subsets are obtained by a traversal on *T*. Additionally, their union must comprise the set of all taxa in the subtree of *T* rooted at $v_{0}$, since $X_{0} \cup \ldots \cup X_\ell$ is simply a partition of all taxa rooted under $v_{0}$.
3. It holds that $C^+_i \subseteq [n] \setminus C^-_0$, since previously-lost characters $C^-_0$ cannot be regained in any $T_{i}$ where $i \in [\ell ]$.
4. By construction, $T_0 \oslash C_0$ is a rooted, node-labeled tree. Since this tree is formed precisely by the traversal from node $v_0$ to the first encountered branching point $v_*$ of *T* without traversing any children of $v_*$, $T_0 \oslash C_0$ itself has no branching points and is thus linear. Since $T_0$ is rooted at $b_0$, $T_0 \oslash C_0$ must be rooted at $b_0 \oslash C_0$.
5. By construction, $T_i \oslash C_i$ is a rooted, node-labeled tree. Since $T_i$ is formed precisely by the traversal of *T* along the *i*-th outgoing edge from node $v_0$ labeled by $b_0$, $T_i \oslash C_{i}$ is rooted at $b_0 \oslash C_{i}$.

□

### Lemma 5

Let *T* be a 1-Dollo phylogeny for matrix *B*. For any node $v_0$ labeled by $b_0$ and descendant node $v_*$ labeled by $b_*$ it holds that $b_* = b(b_0,C^+,C^-)$ where $C^+$ and $C^-$ are the characters that are gained and lost, respectively, on the path from $v_0$ to $v_*$.

### Proof of Lemma 5

This follows directly from Definition 1. □

### Lemma 6

Let $\bar{B}_i$ be a submatrix of $\bar{B}(B,b_*,X_0,C_*)$ defined by characters $C_i \subseteq C_*$ and taxa $X_i \subseteq [m] \setminus X_0$ and let $B_i = B[X_i,C_i]$. Then, character $c \in C_i$ is variable w.r.t. $(B_i,b_*)$ if and only if $\bar{B}_i$ contains a 1 in column *c*.

### Proof of Lemma 6

(⇒) Given binary matrices $\bar{B}_i$ and $B_i$, consider some variable character $c \in C_i$ w.r.t. $B_i$ and $b_{*}$. Therefore, there is some taxon $t \in X_i$ such that $b_{t, c} \ne {b_{*,c}}$. We distinguish two cases.

1. If $b_{*,c} = 0$, then $b_{t, c} = 1$. Thus, $\bar{b}_{t, c} = 1$ by Definition 11.
2. If $b_{*,c} = 1$, then $b_{t, c} = 0$. Thus, $\bar{b}_{t, c} = 1$ by Definition 11.

(⇐) Given binary matrices $\bar{B}_i$ and $B_i$, consider some character $c \in C_i$ such that $\bar{B}_{i}$ contains a 1 in column *c*, that is, there is some taxon $t \in X_{i}$ such that $\bar{b}_{t, c} = 1$. We distinguish two cases.

1. If $b_{*,c} = 0$, then $b_{t, c} = 1$. Thus, $b_{*,c} \ne b_{t, c}$, so *c* is variable w.r.t. $B_i$ and $b_{*}$.
2. If $b_{*,c} = 1$, then $b_{t, c} = 0$. Thus, $b_{*,c} \ne b_{t, c}$, so *c* is variable w.r.t. $B_i$ and $b_{*}$.

□

### Theorem 2

Given matrix $B \in \{0,1\}^{m \times n}$ and binary vector $b_0$, there exists a 1-Dollo phylogeny *T* for *B* rooted at $b_0$ if and only if there exists some set $X_{0} \subseteq [m]$ of taxa and sets $C^-_{0}, C^+_{0} \subseteq [n]$ of characters subject to the following conditions:

1. Sets $X_0$, $C^+_0$, $C^-_0$ are in agreement with *B* and $b_0$.
2. For the 1-Dollo matrix decomposition of *B* and $b_0$ on $X_0$, $C^+_0$, $C^-_0$ into submatrices $\{B_{0} = B[X_{0}, C_{0}], \ldots , B_{\ell } = B[X_{\ell }, C_{\ell }]\}$, there exists a 1-Dollo linear phylogeny $T_0$ for $B_0$ rooted at $b_0 \oslash C_0$ and terminating on $b_{*} \oslash C_0$ and 1-Dollo phylogenies $T_1,\ldots ,T_\ell$ for $B_1,\ldots ,B_\ell$ rooted at $b(b_0, C^+_0, C^-_0) \oslash C_1,\ldots ,b(b_0, C^+_0, C^-_0)\oslash C_\ell$, respectively.

### Proof of Theorem 2

(⇒) Consider a compact 1-Dollo phylogeny *T* for matrix $B \in \{0,1\}^{m \times n}$ rooted at $b_0$. Let $T_0$ be the subtree of *T* obtained by traversing from root node $v_0$ labeled by $b_0$ to a first branching point $v_*$ with label $b_*$, and let $C^+_0$, $C^-_0$ and $X_0$ be the gained characters, lost characters and observed taxa, respectively, in tree $T_0$. Since every character not gained or lost in $T_0$ can never change across the taxa in $X_0$, sets $X_0$, $C^+_0$, $C^-_0$ must be in agreement with *B* and $b_0$. So Condition 1 holds.

Let $\{B_{0}, B_{1}, \ldots , B_{\ell }\}$ be the 1-Dollo matrix decomposition of *B* and $b_{0}$ on $X_{0}$, $C^+_{0}$, $C^-_{0}$. By Lemma 4, then, the tree induced on *T* by taxa set $X_{0}$ is a 1-Dollo linear phylogeny $T_0$ for $B_{0}$ rooted at $b_0$ and terminating at $b_{*}$.

We must finally show that there exist 1-Dollo phylogenies $T_1, \ldots , T_\ell$, for $B_1, \ldots , B_\ell$, respectively rooted at $b_{*} = b(b_{0},C^+_0, C^-_0)$. However, consider the existing subtrees $T_1,\ldots ,T_\ell$ of *T* obtained by traversing along each of the ℓ>1 outgoing edges from $v_*$ indexed by $j \in [\ell ]$, and let $C^+_j$, $C^-_j$ and $X_j$ be the gained characters, lost characters and observed taxa, respectively, in each tree $T_j$. By Lemma 4, these trees are already exactly 1-Dollo phylogenies for $B[X_j, C_j]$ rooted at $b_{*}$. So we will show that for every $i \in [\ell ]$, there is some value of *j*, with existing tree $T_j$, such that $B_{i} = B[X_j, C_j]$. To do this, we will show outright that $\{B_1, \ldots , B_\ell \} = \{B[X_1, C_1], \ldots , B[X_\ell , C_\ell ]\}$.

By Lemma 4, $$\{C_{1},\ldots ,C_{\ell }\}$$ are a partition of characters $$C_* = [n] \setminus C^-_{0}$$ and $$\{X_0,\ldots ,X_\ell \}$$ are a partition of taxa $$[m] \setminus X_0$$. Since *T* is a 1-Dollo phylogeny for *B*, every character $$c \in C_j$$ is variable w.r.t. $$B_{j}$$ and $$b_*$$ and not variable with respect to $$B_{j'}$$ and $$b_*$$ for $$j' \in [\ell ]$$ such that $j\neq j^{\prime }$. So by Lemma 6 and the definition of a complement matrix, $$\{X_{1}, \ldots , X_{\ell }\}$$ and $$\{C_{1},\ldots , C_{\ell }\}$$ are precisely a block diagonal decomposition of $$\bar{B}(B,b_*,X_0,C_*)$$.

Additionally, $\{X_{1}, \ldots , X_{\ell }\}$ and $\{C_{1},\ldots , C_{\ell }\}$ must be such a block diagonal matrix decomposition of maximum size. We will prove this by contradiction. If $\{X_{1}, \ldots , X_{\ell }\}$ and $\{C_{1},\ldots , C_{\ell }\}$ was not on maximum size, it would be the case that for some $B[X_j, C_j]$, that there would exist two submatrices $B[X'_j, C'_j]$ and $B[X''_j, C''_j]$ such that $X'_j$ and $X''_j$ partition $X_j$, $C'_j$ and $C''_j$ partition $C_j$, all characters $c \in C'_j$ were not variable w.r.t. $B[X''_j, C'_j]$ and $b_*$, and all characters $c \in C''_j$ were not variable w.r.t. $B[X'_j, C''_j]$ and $b_*$. This implies that there exists two subtrees $T'_{j}$ and $T''_{j}$ of $T_{j}$ such that $T'_{j}$ contains all taxa in $X'_{j}$ and all gain or loss edges of $C'_j$, $T''_{j}$ contains all taxa in $X''_{j}$ and all gain or loss edges of $C''_j$, and $T'_{j}$ and $T''_{j}$ are disjoint from each other. But then, this implies that edge $(v_*,v_{j})$ in *T* from $v_{*}$ to the root node $v_{j}$ of $T_{j}$ is not a gain or loss edge for any character c∈[n]. Since *T* is compact, this cannot be the case.

By the definition of a 1-Dollo decomposition, the sets of taxa and characters defining $B_1, \ldots , B_\ell$ also comprise a block diagonal decomposition of $\bar{B}(B,b_*,X_0,C_*)$ of maximum size. But the block diagonal matrix decomposition of maximum size for any matrix is unique, so it must be true that $\{B_1, \ldots , B_\ell \} = \{B[X_1, C_1], \ldots , B[X_\ell , C_\ell ]\}$. So for every submatrix $B_i$, there exists a 1-Dollo phylogeny $T_j$ for $B_{i}$ rooted at $b_{*}$. So Condition 2 holds.

(⇐) Given binary matrix *B* and binary vector $b_0$, let there exist $X_0 \subseteq [m]$ and $C^-_0, C^+_0 \subseteq [n]$ such that (i) $X_0$, $C^-_0$, and $C^+_0$ are in agreement with *B* and $b_0$ and (ii) the 1-Dollo matrix decomposition of *B* and $b_0$ on $X_{0}$, $C^+_{0}$, $C^-_{0}$ yields $\{B_{0} = B[X_{0}, C_{0}], B_{1} = B[X_{1}, C_{1}], \ldots , B_{\ell } = B[X_{\ell }, C_{\ell }]\}$ such that there exists 1-Dollo linear phylogeny $T_0$ for $B_0$ rooted at $b_0 \oslash C_{0}$ and terminating on $b_{*} \oslash C_{0}$ and 1-Dollo phylogenies $T_1, \ldots , T_\ell$ for $B_1, \ldots , B_\ell$, respectively, rooted at $b_{*} \oslash C_i$ for $b_{*} = b(b_0, C^+_0, C^-_0)$.

We will construct *T*. Let node $v_{*0}$ be the node of $T_{0}$ labeled by $b_{*} \oslash C_{0}$, and let $v_{*i}$ for all $i \in [\ell ]$ be the root node of $T_{i}$ labeled by $b_{*} \oslash C_{i}$. Then, add edge $(v_{*0}, v_{*i})$ for all $i \in \ell$ to the composite of $T_{0} \oplus b_{0}, T_{1} \oplus b_{*}, \ldots , T_{\ell } \oplus b_{*}$, and subsequently contract all such edges.

Each node of *T* is clearly labeled, and the root of *T* is labeled by $b_{0} = (b_{0} \oslash C_{0}) \oplus b_{0}$, so clearly *T* is rooted at $b_{0}$. First, we prove that for every character *c* such that $b_{0,c} = 1$, there must be no gain edge on *c* in *T*. There is no gain edge for *c* in *T* prior to $v_{*}$, since $T_{0}$ has no gain edge for *c* by definition of rooted 1-Dollo linear phylogeny rooted at $b_{0}$. To demonstrate that there is no gain edge for *c* in *T* after $v_{*}$, we differentiate two cases:

1. $b_{*,c} = 1$. Then, for all $i \in [\ell ], T_{i}$ has no gain edge for *c* by definition of rooted 1-Dollo phylogeny rooted at $b_{*}$. So there is no gain edge for *c* in *T* after encountering $v_{*}$, either, by construction of *T*.
2. $b_{*,c} = 0$. Then, it must be the case that $c \in C^-_0$. But since $C^+_i \subseteq [n] \setminus C^-_0$, $T_{i}$ has no gain edge for *c* for all $i \in [\ell ]$. So there is no gain edge for *c* in *T* after encountering $v_{*}$, either, by construction of *T*.

Second, we prove that for every character *c* such that $b_{0,c} = 1$, there must be at most one loss edge on *c* in *T*. To demonstrate that there is at most one loss edge for *c* in *T* after encountering $v_{*}$, we differentiate two cases:

1. $b_{*,c} = 1$. Then, clearly *c* was not lost in $T_{0}$. By the definition of a block diagonal decomposition, we know that *c* must be variable w.r.t. $B_{i}$ and $b_{*}$ for at most one value of $i \in [\ell ]$. So there must be at most one loss edge in $T_{1}, \ldots , T_{\ell }$.
2. $b_{*,c} = 0$. Then, *c* must have been lost in $T_{0}$. But since $C^+_i \subseteq [n] \setminus C^-_0$, $T_{i}$ has no gain edge, and thus no loss edge, on *c* for all $i \in [\ell ]$.

Third, we prove that for every character *c* such that $b_{0,c} = 0$, there must be at most one gain and at most one loss edge on *c* in *T*. We prove three statements that together demonstrate this in full:

1. For any character *c*, there cannot be a gain edge in $T_{0}$ and a gain edge in $T_{i}$ for $i \in [\ell ]$. If there was, then $c \in C^-_0$. But since $C^+_i \subseteq [n] \setminus C^-_0$, so $T_{i}$, cannot have a gain edge, and thus cannot have a loss edge, on *c* for all $i \in [\ell ]$.
2. For any character *c*, there cannot be a loss edge in $T_{0}$ and a loss edge in $T_{i}$ for $i \in [\ell ]$. If there was, then $c \in C^-_0$. But since $C^+_i \subseteq [n] \setminus C^-_0$, $T_{i}$ cannot have a gain edge, and thus cannot have a loss edge, on *c* for all $i \in [\ell ]$.
3. For any character *c*, there cannot be a gain edge in both $T_{i}$ and $T_{j}$ for distinct $i, j \in [\ell ]$ such that i≠j. This follows from the definition of a block matrix decomposition, since *c* must be variable w.r.t. $B_{i}$ and $b_{*}$ for at most one value of $i \in [\ell ]$.

So *T* is a valid 1-Dollo phylogeny for *B*, and we are done. □
